# lncRNA H19 mediates BMP9-induced osteogenic differentiation of mesenchymal stem cells (MSCs) through Notch signaling

**DOI:** 10.18632/oncotarget.18655

**Published:** 2017-06-27

**Authors:** Junyi Liao, Xinyi Yu, Xue Hu, Jiaming Fan, Jing Wang, Zhicai Zhang, Chen Zhao, Zongyue Zeng, Yi Shu, Ruyi Zhang, Shujuan Yan, Yasha Li, Wenwen Zhang, Jing Cui, Chao Ma, Li Li, Yichun Yu, Tingting Wu, Xingye Wu, Jiayan Lei, Jia Wang, Chao Yang, Ke Wu, Ying Wu, Jun Tang, Bai-Cheng He, Zhong-Liang Deng, Hue H. Luu, Rex C. Haydon, Russell R. Reid, Michael J. Lee, Jennifer Moriatis Wolf, Wei Huang, Tong-Chuan He

**Affiliations:** ^1^ Departments of Orthopaedic Surgery, Blood Transfusion, Nephrology, and General Surgery, The First Affiliated Hospital of Chongqing Medical University, Chongqing, China; ^2^ Molecular Oncology Laboratory, Department of Orthopaedic Surgery and Rehabilitation Medicine, The University of Chicago Medical Center, Chicago, IL, USA; ^3^ Ministry of Education Key Laboratory of Diagnostic Medicine, and The Affiliated Hospitals of Chongqing Medical University, Chongqing, China; ^4^ Department of Orthopaedic Surgery, Union Hospital of Tongji Medical College, Huazhong University of Science & Technology, Wuhan, China; ^5^ Department of Laboratory Medicine and Clinical Diagnostics, The Affiliated Yantai Hospital, Binzhou Medical University, Yantai, China; ^6^ Departments of Neurosurgery, and Otolaryngology-Head & Neck Surgery, The Affiliated Zhongnan Hospital of Wuhan University, Wuhan, China; ^7^ Department of Biomedical Engineering, School of Bioengineering, Chongqing University, Chongqing, China; ^8^ Department of Emergency Medicine, Beijing Hospital, Beijing, China; ^9^ Department of Immunology and Microbiology, Beijing University of Chinese Medicine, Beijing, China; ^10^ Cytate Institute for Precision Medicine & Innovation, Guangzhou Cytate Biomedical Technologies Inc., Guangzhou, China; ^11^ Department of Surgery, Section of Plastic Surgery, The University of Chicago Medical Center, Chicago, IL, USA

**Keywords:** mesenchymal stem cells, BMP9, osteogenic differentiation, lncRNA H19, Notch signaling

## Abstract

Mesenchymal stem cells (MSCs) are multipotent progenitor cells that can undergo self-renewal and differentiate into multiple lineages. Osteogenic differentiation from MSCs is a well-orchestrated process and regulated by multiple signaling pathways. We previously demonstrated that BMP9 is one of the most potent osteogenic factors. However, molecular mechanism through which BMP9 governs osteoblastic differentiation remains to be fully understood. Increasing evidence indicates noncoding RNAs (ncRNAs) may play important regulatory roles in many physiological and/or pathologic processes. In this study, we investigate the role of lncRNA H19 in BMP9-regulated osteogenic differentiation of MSCs. We demonstrated that H19 was sharply upregulated at the early stage of BMP9 stimulation of MSCs, followed by a rapid decease and gradual return to basal level. This process was correlated with BMP9-induced expression of osteogenic markers. Interestingly, either constitutive H19 expression or silencing H19 expression in MSCs significantly impaired BMP9-induced osteogenic differentiation *in vitro* and *in vivo*, which was effectively rescued by the activation of Notch signaling. Either constitutive H19 expression or silencing H19 expression led to the increased expression of a group of miRNAs that are predicted to target Notch ligands and receptors. Thus, these results indicate that lncRNA H19 functions as an important mediator of BMP9 signaling by modulating Notch signaling-targeting miRNAs. Our findings suggest that the well-coordinated biphasic expression of lncRNA H19 may be essential in BMP9-induced osteogenic differentiation of MSCs, and that dysregulated H19 expression may impair normal osteogenesis, leading to pathogenic processes, such as bone tumor development.

## INTRODUCTION

Mesenchymal stem cells (MSCs) are multipotent progenitor cells that can undergo self-renewal and differentiate into multiple lineages, including osteoblastic, chondrogenic, and adipogenic lineages [1–6]. Owing to their broad range utilities and easiness of manipulations, MSCs attract profound attention in the arena of stem cell biology and regenerative medicine [4, 7–10]. On the other hand, osteogenic differentiation from MSCs occurs via the sequential events that recapitulate most of the molecular and cellular processes occurring during bone and skeletal development [11].

While several signaling pathways, such as Wnt, Insulin-like growth factors (IGFs), Fibroblast growth factor (FGFs) and Notch, play important roles in regulating osteogenic differentiation [3, 12–21], bone morphogenetic proteins (BMPs) are considered the most potent osteoinductive factors [22, 23]. BMPs belong to the TGF-β superfamily and play critical roles in skeletal development, bone formation and stem cell differentiation [3, 22–24]. At least 15 different BMPs have been identified in humans and rodents, and disruptions in BMP signaling in mice result in a variety of skeletal and extraskeletal anomalies [22, 23, 25]. Through a comprehensive analysis of the 14 types of BMPs’ osteogenic activities, we found that BMP9 (also known as growth differentiation factor 2, or GDF2) is among the most osteogenic BMPs that induce osteoblastic differentiation of MSCs [22, 26–29]. We demonstrated that BMP9 is resistant to naturally occurring antagonist noggin, which may at least partially contribute to its potent osteogenic activity [30]. We further demonstrated that TGFβ/BMP type I receptors ALK1 and ALK2 are essential for BMP9-induced osteogenic signaling in MSCs [31]. Nonetheless, the exact signaling mechanisms through which BMP9 regulates osteogenic differentiation remain to be fully understood.

Increasing evidence indicates noncoding RNAs (ncRNAs) may play essential regulatory roles in many physiological and/or pathologic processes [32–41]. While over80% of the human genome is transcribed, it is currently estimated that <2% of the human genome is transcribed into mRNA, leaving most of the transcripts as ncRNAs [36–40, 42]. The past several years have seen a huge expansion of our knowledge about the important regulatory functions of ncRNAs, especially long noncoding RNAs (lncRNAs).

In this study, we investigate the possible role of lncRNA H19 in BMP9-regulated osteogenic differentiation of MSCs. Identified as one of the first imprinted genes and ncRNAs, H19 is known to participate in highly diverse cellular processes including tumorigenesis, control of embryonic growth and stem cell differentiation [43–47]. We found that H19 was sharply upregulated at the early stage of BMP9 stimulation of MSCs, followed by a rapid decrease to a low level and gradual return to near basal level. This process correlated with the BMP9-induced expression of osteogenic markers. Either a constitutive expression of H19 or silencing H19 expression significantly impaired BMP9-induced osteogenic differentiation *in vitro* and *in vivo*, which was effectively rescued by activating Notch signaling. Further analysis revealed that a constitutive expression of H19 or silencing H19 expression resulted in the increased expression of a group of microRNAs (miRNAs) that target Notch ligands and receptors. Thus, our results demonstrate that lncRNA H19 is an important mediator of BMP9 signaling, suggesting that a balanced lncRNA H19 expression may play an essential role in BMP9-induced osteogenic differentiation of MSCs, and that disruptions of H19 expression may impair normal osteogenesis, leading to pathogenic processes such as bone tumor development.

## RESULTS

### BMP9 induces biphasic expression of lncRNA H19, which correlates with the expression of osteogenic regulators and late osteogenic markers in MSCs

**Figure 1:**
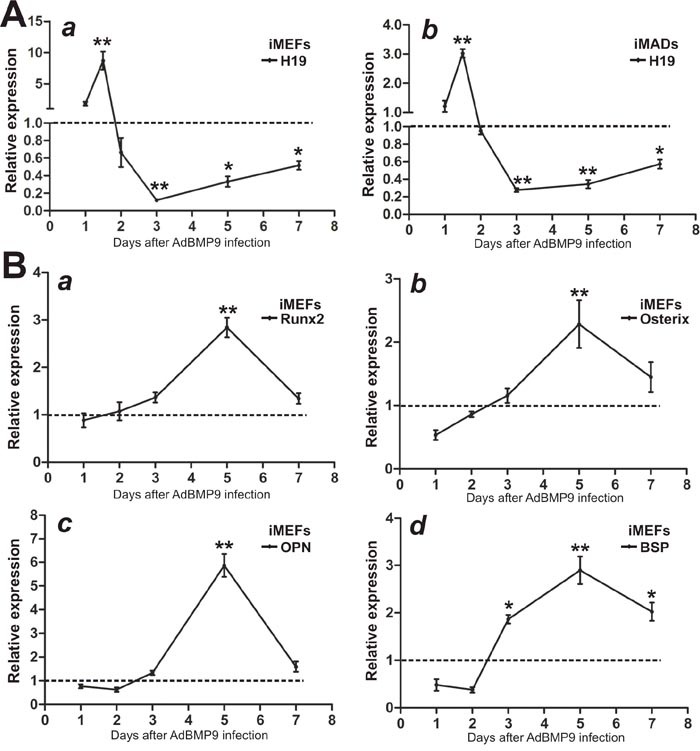
Expression levels of lncRNA H19 and osteogenic markers in BMP9-induced osteogenic differentiation of MSCs Exponentially growing iMEFs or iMADs were infected with AdBMP9 or AdGFP. At the indicated time points, total RNA was isolated and subjected to TqPCR analysis using PCR primers specific for mouse H19 **(A)** and other osteogenic markers **(B)**. All samples were normalized with the reference gene *Gapdh*. Each assay condition was done in triplicate. Relative expression was calculated by dividing the relative expression values (i.e., gene/Gapdh) in BMP9-treated group with that from the GFP group. “**” p < 0.001, “*” p < 0.05, AdBMP9 group vs. AdGFP group. Runx2, runt-related transcription factor 2; OPN, osteopontin; BSP, bone sialoprotein.

Considering the important roles that lncRNA H19 in development and stem cell differentiation, we examined the expression patterns of H19 in MSCs upon BMP9 stimulation. Using the previously well-characterized MSC lines iMEFs and iMADs [48–50], we found that at the early stage of BMP9 stimulation (24h and 36h), the expression of H19 increased dramatically and reached a peak at 36h in both iMEFs and iMADs cells, compared with that of the control group (Figure 1A–ab). However, H19 expression decreased rather rapidly at 48h and reached the lowest level at 72h after BMP9 stimulation in both MSC lines. Nonetheless, H19 expression recovered gradually from days 3 to 7 after BMP9 stimulation in both iMEFs and iMADs cells (Figure 1A). These results suggest that a balanced, biphasic expression of lncRNA H19 may play an important role in BMP9-induced osteogenic differentiation of MSCs.

We further analyzed whether the expression of osteogenic regulators and osteogenic markers correlated with the H19 expression pattern. We found that both osteogenic lineage regulators Runx2 and Osterix were up-regulated starting at day 3 after BMP9 simulation and peaked at day 5 (Figure 1B–ab), trailing the early increase of H19 expression but correlating with the recovery of H19 expression at later time points. Accordingly, the expression of late osteogenic markers OPN and BSP was shown to increase at 3 days after BMP9 stimulation (Figure 1B–cd). Taken together, these results indicate that H19 may affect the early stage of BMP9-induced osteogenic differentiation, preceding the full commitment to osteogenic lineage of MSCs.

### Silencing lncRNA H19 expression diminishes BMP9-induced osteogenic differentiation of MSCs *in vitro*

**Figure 2:**
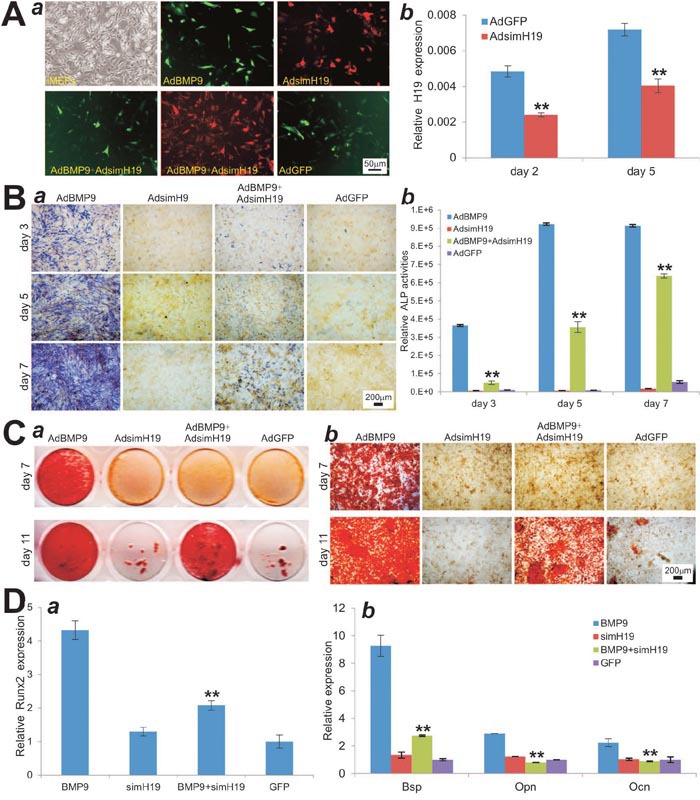
Silencing lncRNA H19 expression diminishes BMP9-induced osteogenic differentiation of MSCs *in vitro* **(A)** Effective knockdown of mouse H19 expression. (***a***) The AdsimH19 expressing siRNA targeting mouse H19 transduces iMEF cells with high efficiency in single or combination infection. (***b***) AdsimH19 silences the expression of H19 at day 2 and day 5. All samples were normalized with the reference gene Gapdh. Each assay condition was done in triplicate. “**” p < 0.001, AdsimH19 *vs*. AdGFP groups. **(B)** AdsimH19 inhibits BMP9-induced ALP activity in MSCs. Subconfluent iMEFs were infected with AdBMP9 or AdGFP and/or AdsimH19. At the indicated time points, the infected cells were subjected to ALP activity assays by either histochemical staining (***a***) or quantitative bioluminescence assay (***b***). Each assay conditions were done in triplicate. Representative staining is shown. “**” p < 0.001, AdBMP9 group *vs*. AdBMP9+AdsimH19 group. **(C)** AdsimH19 inhibits BMP9-induced calcium deposit. Subconfluent iMEFs were infected with AdBMP9 or AdGFP and/or AdsimH19, and cultured in mineralization medium. At the indicated time points, the infected cells were fixed and subjected to Alizarin Red S staining. Each assay condition was done in triplicate. Representative gross images (***a***) and microscopic images (20x) (***b***) are shown. **(D)** AdsimH19 inhibits BMP9-induced osteogenic regulator Runx2 at day 3 (***a***) and late osteogenic differentiation marker Bsp, Opn and Ocn at day 5 (***b***). “**” p < 0.001, AdBMP9 group vs. AdBMP9+AdsimH19 group.

To effectively knock down H19 expression in MSCs, we constructed an adenoviral vector expressing three siRNA sites targeting mouse H19. We showed that AdsimH19 alone or with AdBMP9 can effectively infect iMEFs (Figure 2A–a). Using TqPCR analysis [51], we demonstrated that at both days 2 and 5, AdsimH19 was able to effectively downregulate the expression of H19 (Figure 2A–b).

We analyzed the effect of down-regulation of H19 expression on BMP9-induced osteogenic differentiation of MSCs. When iMEFs were co-infected with AdBMP9 and AdsimH19, we found BMP9-induced ALP activities were significantly inhibited at days 3 and 5, and to lesser extents at day 7, both qualitatively and quantitatively (Figure 2B–ab). Similarly, BMP9-induced matrix mineralization in iMEFs was significantly inhibited at days 7 and 11 when the expression of H19 was silenced (Figure 2C–ab). When the expression of osteogenic regulators and markers were assessed, we found that BMP9-induced expression of Runx2 in iMEFs was dramatically reduced by AdsimH19 co-infection (Figure 2D–a). Accordingly, silencing H19 expression in iMEFs led to a significant decrease in the expression of late osteogenic markers such as Bsp, Opn and Ocn at day 5 (Figure 2D–b). Taken together, these results indicate that silencing H19 expression can significantly diminish BMP9-induced osteogenic differentiation, suggesting that H19 may play an essential role in BMP9-induced osteogenic differentiation.

### Constitutive expression of lncRNA H19 blocks BMP9-induced osteogenic differentiation of MSCs *in vitro*

**Figure 3:**
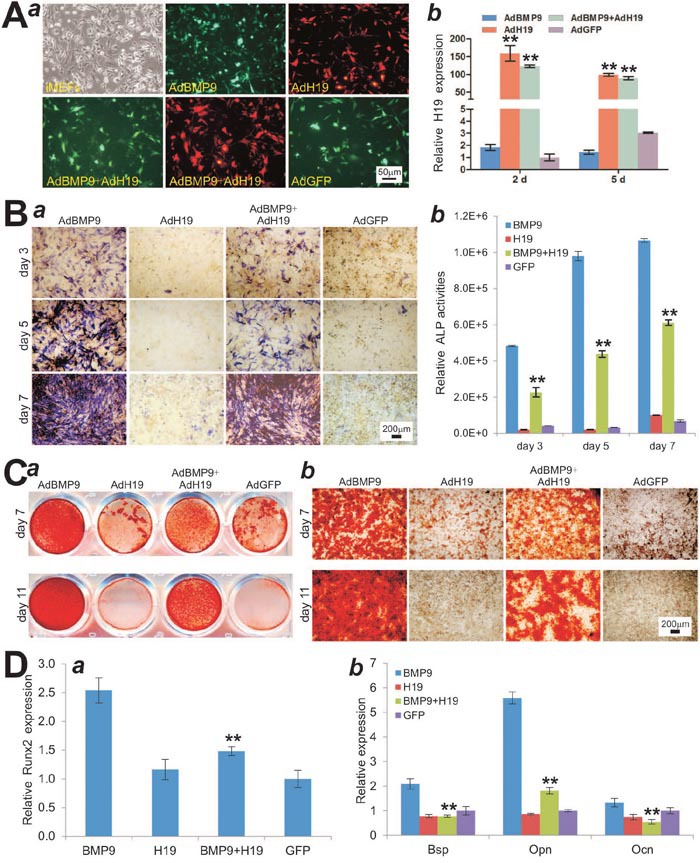
Overexpression of lncRNA H19 blocks BMP9-induced osteogenic differentiation of MSCs *in vitro* **(A)** Adenovirus AdH19-mediated overexpression of mouse H19. (***a***) AdH19 transduces iMEF cells with high efficiency. (***b***) AdH19 increases the expression of H19 more than 50 times at day 2 and day 5. All samples were normalized with the reference gene *Gapdh*. Each assay condition was done in triplicate. “**” p < 0.001, AdH19 group vs. AdGFP group. **(B)** Overexpression of H19 inhibits BMP9-induced ALP activity in MSCs. Subconfluent iMEFs were infected with AdBMP9 or AdGFP and/or AdH19. At the indicated time points, the infected cells were subjected to ALP activity assays by either histochemical staining (***a***) or quantitative bioluminescence assay (***b***). Each assay condition was done in triplicate. Representative staining is shown. “**” p < 0.001, AdBMP9 group vs. AdBMP9+AdH19 group. **(C)** Overexpression of H19 inhibits BMP9-induced calcium deposit. Subconfluent iMEFs were infected with AdBMP9 or AdGFP and/or AdH19, and cultured in mineralization medium. At the indicated time points, the infected cells were fixed and subjected to Alizarin Red S staining. Each assay condition was done in triplicate. Representative gross images (***a***) and microscopic images (20x) (***b***) are shown. **(D)** Overexpression of H19 inhibits BMP9-induced early osteogenic regulator Runx2 at day 3 (***a***) and late osteogenic differentiation marker Bsp, Opn and Ocn at day 5 (***b***). “**” p < 0.001, AdBMP9 group *vs*. AdBMP9+AdH19 group.

We further determined the effect of H19 overexpression on BMP9-induced osteogenic differentiation of MSCs. To effectively overexpress H19, we constructed an adenoviral vector AdH19 and showed that AdH19 infected the iMEF cells with high efficiency (Figure 3A–a). Quantitative PCR analysis indicated that AdH19-mediated expression of H19 was more than 50 fold higher than that of the control group (Figure 3A–b). When iMEFs were co-infected with AdBMP9 and AdH19, we found BMP9-induced ALP activity was significantly inhibited at days 3 and 5, and to lesser extents at day 7 by ALP staining(Figure 3B–a). Quantitatively, overexpression of H19 in iMEFs led to a decreased ALP activity to 46.9%, 44.7% and 57.3% of that for AdBMP9 alone at days 3, 5, and 7, respectively (Figure 3B–b). Furthermore, we found that BMP9-induced matrix mineralization in iMEFs was significantly inhibited by H19 overexpression at days 7 and 11 (Figure 3C–ab). Similarly, we found that BMP9-induced expression of osteogenic transcription regulator Runx2 was significantly decreased when H19 was co-expressed (Figure 3D–a), and overexpression of H19 also inhibited BMP9-induced expression of late osteogenic markers, such as Bsp, Opn and Ocn (Figure 3D–b). Taken together, these results indicate that the overexpression of H19 can block BMP9-induced osteogenic differentiation *in vitro*, suggesting that maintaining appropriate expression level of H19 may be critical for BMP9-induced osteogenic differentiation of MSCs.

### Both overexpression and silencing of lncRNA H19 inhibit the terminal differentiation of BMP9-induced ectopic bone formation from MSCs

**Figure 4:**
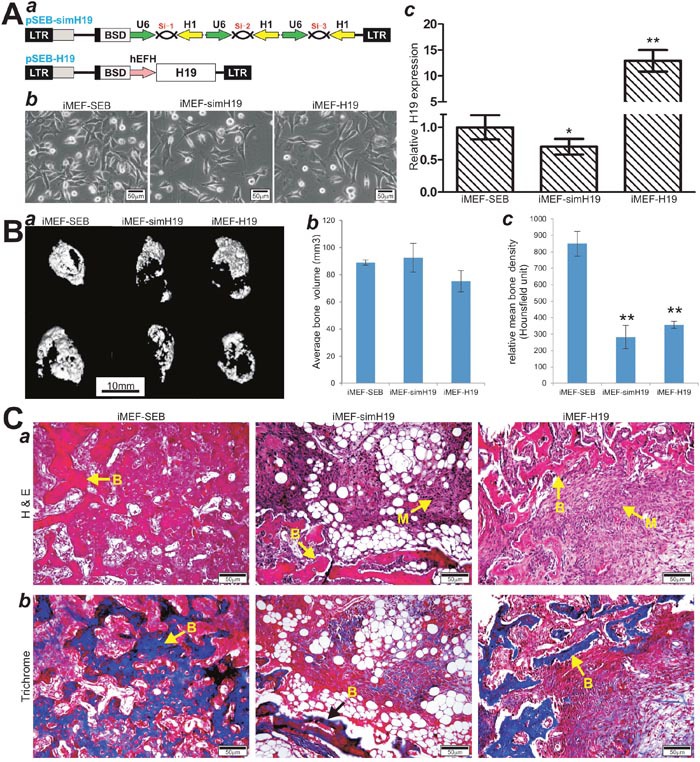
Stable overexpression and silencing of lncRNA H19 inhibit BMP9-induced ectopic bone formation *in vivo* **(A)** Stable expression of mouse H19 or siRNAs silencing H19 in MSCs. (***a***) Schematic representation of the retroviral vectors expressing siRNAs targeting mouse H19 or the full-length transcript of mouse H19 (***a***). The retroviral vectors also express blasticidin resistance gene. (***b***) The stable iMEFs lines that express simH19 (iMEF-simH19), mouse H19 (iMEF-H19), or empty vector (iMEF-SEB). (***c***) The expression levels of H19 in the three stable lines were assessed by TqPCR, “*” p < 0.05, iMEF-SEB group vs. iMEF-simH19 group; “**”, p < 0.001 iMEF-SEB group *vs*. iMEF-H19 group. **(B)** μCT imaging of the BMP9-induced ectopic bone formation of the three stable lines. (***a***) μCT imaging and 3D reconstruction of the retrieved bone masses from the indicated groups at 4 weeks **(a)**. No significant volumetric differences were found among the three groups (***b***) (*p>0.05*). Mean bone density was analyzed in each group. “**” p < 0.001, iMEF-SEB group vs. iMEF-simH19 or iMEF-H19 group. **(C)** H & E (***a***) and Masson’s Trichrome staining (***b***) of the bone masses retrieved at week 4. Representative images are shown. **(B)** mature bone; M, undifferentiated MSCs.

To further assess the *in vivo* role of lncRNA H19 in BMP9-induced osteogenesis, we constructed a retroviral vector system to stably overexpress H19 or siRNAs that target mouse H19 (simH19) (Figure 4A–a). Using these retroviral vectors, we established three iMEF lines that stably express mouse H19 (iMEF-H19), simH19 (iMEF-simH19), and vector only (iMEF-SEB), respectively (Figure 4A–b). TqPCR analysis indicated that the expression level of H19 were significantly decreased in the iMEF-simH19 cells (p<0.05) while drastically increased in iMEF-H19 cells (p<0.001), when compared with that of the control iMEF-SEB cells (Figure 4A–c).

Using the stable iMEF lines, we conducted the *in vivo* stem cell implantation studies by transducing the cells with AdBMP9 or AdGFP and subcutaneously injecting the cells into the flanks of athymic nude mice for 4 weeks. Consistent with our previous reports [50], bony masses were retrieved from the injection sites in AdBMP9 treatment groups while no masses were retrieved from the injection sites in the AdGFP only groups. μCT imaging analysis indicated that either overexpression of H19 or silencing H19 expression did not significantly affect the size or volumetric values of ectopic bone masses, compared with that of the iMEF-SEB control group (p>0.05) (Figure 4B–ab). However, the masses retrieved from both iMEF-simH19 and iMEF-H19 groups exhibited significantly lower mean bone mineral density, compared with that of the iMEF-SEB group (*p<0.001*) (Figure 4B–c).

H & E staining of the retrieved masses further confirmed that either silencing H19 expression or overexpression of H19 significantly diminished BMP9-induced bone formation, as evidenced by a decreased formation of trabecular bones and/or osteoid matrix, compared with that of the iMEF-SEB group (Figure 4C–a). Interestingly, silencing H19 expression in iMEFs seemingly led to formation of a significant amount of adipocytes upon BMP9 stimulation (Figure 4C–a), as we previously demonstrated that BMP9 is capable of inducing both osteogenic and adipogenic differentiation of MSCs [28, 29, 52]. Trichrome staining further demonstrated that the masses retrieved from both iMEF-simH19 and iMEF-H19 groups exhibited very little mineralized and mature osteoid matrix, compared with that of the iMEF-SEB group (Figure 4C–b). Collectively, these results strongly suggest that lncRNA H19 may play a delicate role in fine-tune regulation of BMP9-induced osteogenic differentiation of MSCs as either H19 overexpression or silencing H19 expression inhibits the terminal osteogenic differentiation of MSCs initiated by BMP9.

### Osteogenic deficiency in H19-silenced MSCs can be rescued through the activation of the Notch signaling pathway

**Figure 5:**
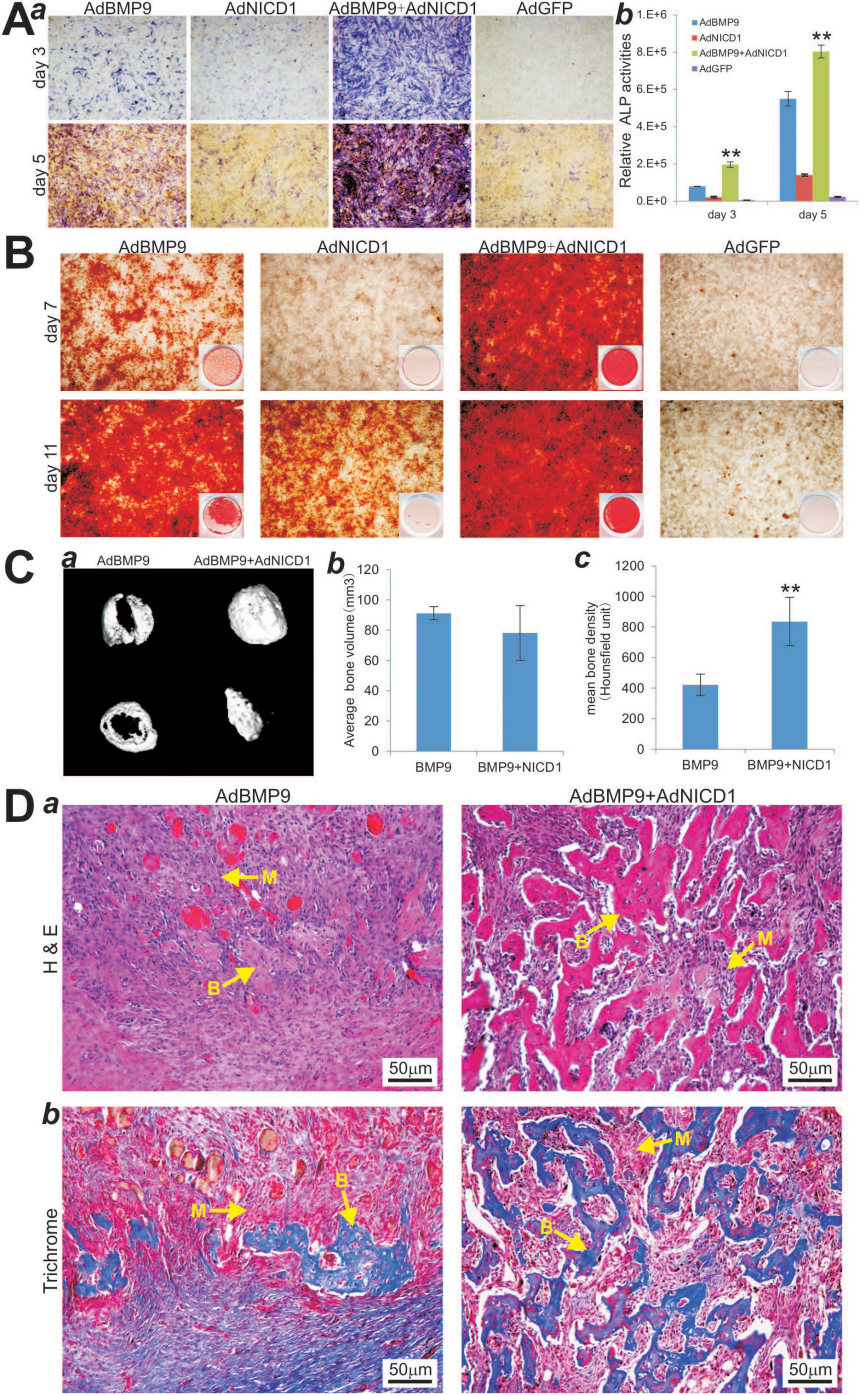
Activation of Notch signaling through exogenous expression of Notch1 introcellular domain (NICD1) restores BMP9-induced osteogenic differentiation in iMEF-simH19 cells **(A)** AdNICD1-mediated expression of NICD1 in iMEF-simH19 cells rescues BMP9-induced ALP activity. Subconfluent iMEF-simH19 cells were infected with AdBMP9, AdNICD1, AdBMP9+AdNCID1 or AdGFP. At the indicated time points ALP activity was determined by qualitative histochemical staining (***a***) and quantitative bioluminescence assay (***b***). “**” p < 0.001, AdBMP9 group vs. AdBMP9+AdNICD1 group. **(B)** NICD1 expression rescues down-regulated calcium deposit in iMEF-simH19 cells. Subconfluent iMEF-simH19 cells were infected with AdBMP9, AdNICD1, AdBMP9+AdNCID1 or AdGFP, and cultured in mineralization medium. At the indicated time points, the infected cells were fixed and subjected to Alizarin Red S staining. Each assay condition was done in triplicate. Representative gross images (***a***) and microscopic images (20x) (***b***) are shown. **(C)** μCT imaging of the BMP9-induced ectopic bone formation in iMEF-simH19 cells that were infected with AdBMP9 or AdBMP9+AdNICD1. (***a***) μCT imaging and 3D reconstruction of the retrieved bone masses from the indicated groups at 4 weeks (***a***). No significant volumetric differences were found among the three groups (***b***) (p>0.05). Mean bone density was analyzed in each group. “**” p < 0.001, AdBMP9 group vs. AdBMP9+AdNICD1 group. **(D)** H & E (***a***) and Masson’s Trichrome staining (***b***) of the bone masses retrieved at week 4. Representative images are shown. B, mature bone; M, undifferentiated MSCs.

Notch signaling is known to play an important role in regulating osteogenic differentiation and bone formation [18, 53, 54]. We tested whether activation of Notch signaling would rescue osteogenic differentiation deficiency of iMEF-simH19 cells upon BMP9 stimulation. To activate the Notch signaling pathway, we previously constructed an adenoviral vector expressing a constitutively active form of Notch1, which contains the Notch1 intracellular domain (NICD1) [55]. When iMEF-simH19 cells were co-infected with AdBMP9 and AdNICD1, we found that BMP9-induced ALP activity was significantly restored, compared with that stimulated with BMP9 alone (Figure 5A–ab). Furthermore, overexpression of NICD1 in iMEF-simH19 cells significantly improved BMP9-induced calcium deposition and matrix mineralization (Figure 5B–ab).

We further tested the *in vivo* effect of NICD1 expression on iMEF-simH19 cells upon BMP9 stimulation. AdNICD1 infected iMEF-simH19 cells were implanted subcutaneously and formed robust bony masses upon BMP9 stimulation, which exhibited no significant volumetric difference compared with the AdBMP9 alone group (p>0.05) (Figure 5C–ab). However, μCT imaging analysis indicated that overexpression of NICD1 in iMEF-simH19 significantly improved mean bone mineral density upon BMP9 stimulation, compared with that AdBMP9 alone group (Figure 5C–c). Histologic analysis revealed that overexpression of NICD1 drastically increased the amount of trabecular bone with significantly fewer undifferentiated cells left in the masses in BMP9-stimulated iMEF-simH19 cells, compared with that of the AdBMP9 alone group (Figure 5D–a). Trichrome staining further confirmed that NICD1 overexpression in iMEF-simH19 cells formed more mature and better mineralized bone matrices, compared with that of AdBMP9 alone group (Figure 5D–b), which was consistent with the results from μCT analysis of the mean mineral density as shown in (Figure 5C-c). Collectively, these *in vivo* results suggest that exogenous expression of NICD1 may rescue the diminished terminal osteogenic differentiation caused by the absence of lncRNA H19 in MSCs.

### Activation of Notch signaling restores the osteogenic differentiation impaired by the constitutive expression of lncRNA H19 in MSCs

**Figure 6:**
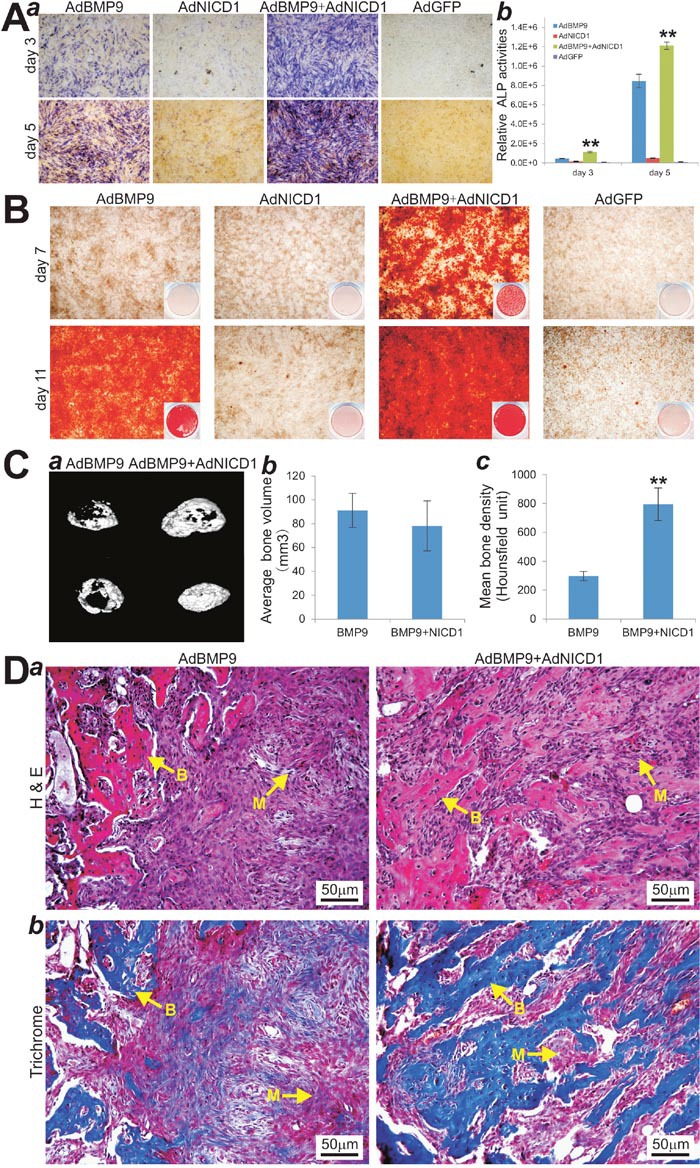
NICD1 expression rescues the deficiency of BMP9-induced osteogenic differentiation in iMEF-H19 cells **(A)** AdNICD1-mediated expression of NICD1 in iMEF-H19 cells rescues BMP9-induced ALP activity. Subconfluent iMEF-H19 cells were infected with AdBMP9, AdNICD1, AdBMP9+AdNCID1 or AdGFP. At the indicated time points ALP activity was determined by qualitative histochemical staining (***a***) and quantitative bioluminescence assay (***b***). “**” p < 0.001, AdBMP9 group vs. AdBMP9+AdNICD1 group. **(B)** NICD1 expression rescues down-regulated calcium deposit in iMEF-H19 cells. Subconfluent iMEF-H19 cells were infected with AdBMP9, AdNICD1, AdBMP9+AdNCID1 or AdGFP, and cultured in mineralization medium. At the indicated time points, the infected cells were fixed and subjected to Alizarin Red S staining. Each assay condition was done in triplicate. Representative gross images (***a***) and microscopic images (20x) (***b***) are shown. **(C)** μCT imaging of the BMP9-induced ectopic bone formation in iMEF-H19 cells that were infected with AdBMP9 or AdBMP9+AdNICD1. (***a***) μCT imaging and 3D reconstruction of the retrieved bone masses from the indicated groups at 4 weeks (***a***). No significant volumetric differences were found among the three groups (***b***) (p>0.05). Mean bone density was analyzed in each group. “**” p < 0.001, AdBMP9 group vs. AdBMP9+AdNICD1 group. **(D)** H & E (***a***) and Masson’s Trichrome staining (***b***) of the bone masses retrieved at week 4. Representative images are shown. B, mature bone; M, undifferentiated MSCs.

We also tested whether activation of Notch signaling would rescue BMP9-induced osteogenic differentiation deficiency of iMEF-H19 cells. When iMEF-H19 cells were co-infected with AdBMP9 and AdNICD1, we found that BMP9-induced ALP activity was significantly enhanced, compared with that stimulated with BMP9 alone (Figure 6A–ab). Overexpression of NICD1 in iMEF-H19 cells significantly improved BMP9-induced calcium deposition and matrix mineralization (Figure 6B–ab).

We studied the *in vivo* effect of NICD1 expression on iMEF-H19 cells upon BMP9 stimulation. We found that subcutaneously implanted AdNICD1 infected iMEF-H19 cells formed robust bony masses upon BMP9 stimulation, which exhibited no significant volumetric difference compared with the AdBMP9 alone group (p>0.05) (Figure 6C–ab). However, μCT imaging analysis revealed that overexpression of NICD1 in iMEF-H19 significantly improved mean bone mineral density upon BMP9 stimulation, compared with that AdBMP9 alone group (Figure 6C–c). Histologic analysis further revealed that overexpression of NICD1 drastically increased the amount of trabecular bone with significantly fewer undifferentiated cells left in the masses in BMP9-stimulated iMEF-H19 cells, compared with that of the AdBMP9 alone group (Figure 6D–a). Trichrome staining further confirmed that NICD1 overexpression in iMEF-H19 cells formed more mature and better mineralized bone matrices, compared with that of AdBMP9 alone group (Figure 6D–b), which was consistent with the results from μCT analysis of the mean mineral density as shown in (Figure 6C-c). Collectively, these *in vivo* results suggest that exogenous expression of NICD1 may rescue the impaired terminal osteogenic differentiation characteristic of constitutive lncRNA H19 expression in MSCs.

### lncRNA H19 mediates BMP9-induced osteogenic differentiation through modulating the expression of Notch ligands

**Figure 7:**
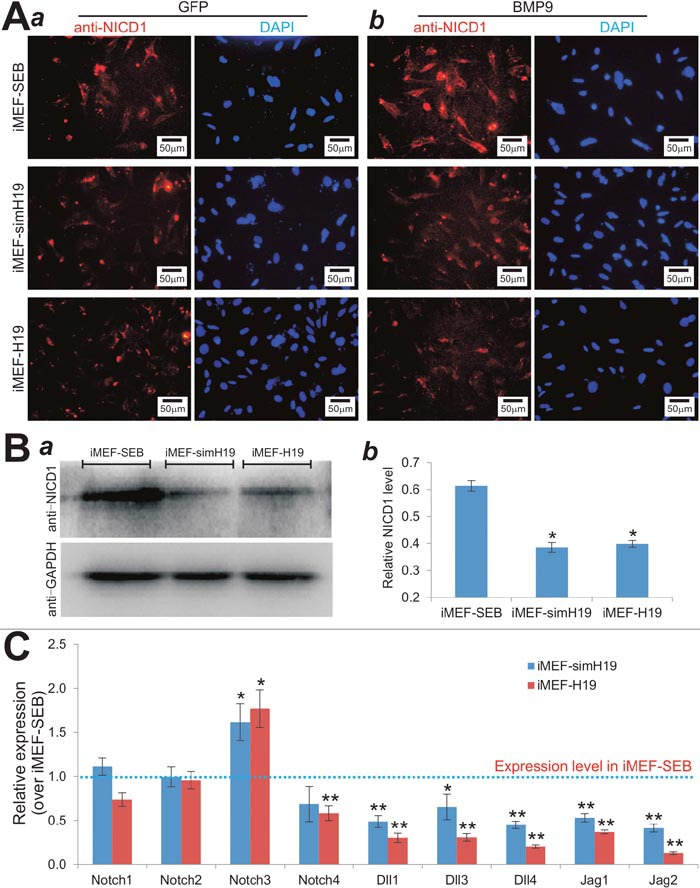
lncRNA H19 mediates BMP9-induced osteogenic differentiation by modulating Notch signaling **(A)** Immunofluorescence staining of NICD1 in H19 silenced or overexpressed MSCs upon BMP9 stimulation. Subconfluent iMEF-SEB, iMEF-simH19 and iMEF-H19 cells were infected with AdGFP (***a***) or AdBMP9 (***b***) for 36h, fixed and subjected to immunofluorescence staining using an NICD1 antibody. Cell nuclei were counterstained with DAPI. No primary antibody was used as a negative control. Each staining condition was done in duplicate. Representative images are shown. **(B)** Western blotting analysis of basal NICD1 levels in H19 silenced or overexpressed MSCs. Exponentially growing iMEF-SEB, iMEF-simH19 and iMEF-H19 cells were lysed in Laemmli sample buffer and subjected to SDS-PAGE and Western blotting analysis using NICD1 or GAPDH antibody. The protein of interest was detected by enhanced chemiluminescence (ECL) (***a***), which was further quantified densitometrically (***b***). “*” p<0.05, compared with that of iMEF-SEB cells. **(C)** Relative expression levels of Notch ligands and receptors in the MSCs in which H19 was silenced or overexpressed. Total RNA was isolated from exponentially growing iMEF-SEB, iMEF-simH19 and iMEF-H19 cells, and subjected to TqPCR using the primers for the indicated genes. *Gapdh* was used as a reference gene. Each qPCR assay condition was done in triplicate. “*” *p<0.05*, “**” *p<0.001*, compared with the relative expression levels in iMEF-SEB cells (the dotted line).

To understand how H19 may influence Notch signaling in MSCs, we examined whether BMP9-induced activation of Notch signaling was impaired when H19 was silenced or overexpressed. Consistent with our earlier findings [55], BMP9 stimulation induced cytoplasmic/nuclear accumulation of NICD in MSCs (Figure 7A, 7Aa *vs*. 7Ab). However, the BMP9-induced NICD accumulation was significantly diminished both in H19 silenced MSCs (iMEF-simH19) and H19 overexpressing MSCs (iMEF-H19) (Figure 7A, 7Aa *vs*. 7Ab). These results were further confirmed by Western blotting analysis, in which endogenous NICD accumulation was significantly less pronounced in both iMEF-simH19 and iMEF-H19 cells, compared with that of the iMEF-SEB cells (Figure 7B–ab). Collectively, these results indicate that lncRNA H19 may influence BMP9 signaling in MSCs by regulating Notch signaling.

We further analyzed the basal expression status of Notch receptors and ligands in MSCs when H19 was silenced or overexpressed. We found that when H19 was silenced or overexpressed, the expression levels of Notch1 and Notch2 did not change significantly, but Notch3 expression was slightly elevated and Notch4 expression was slightly decreased (Figure 7C). However, the expression levels of all Notch ligands (i.e., Dll1, Dll3, Dll4, Jag1 and Jag2) were significantly downregulated both in iMEF-simH19 and iMEF-H19 cells (Figure 7C). Interestingly, under the same assay condition H19 overexpression seemingly led to a greater decrease in the expression of Notch ligands, compared with that of the H19 silenced MSCs (Figure 7C). Taken together, these results suggest that the decreased NICD accumulation in iMEF-simH19 and iMEF-H19 cells may be caused by the decreased levels of Notch ligands.

### Both silencing H19 expression and constitutive H19 expression lead to the increased expression of the miRNAs that target Notch ligands and receptors

**Figure 8:**
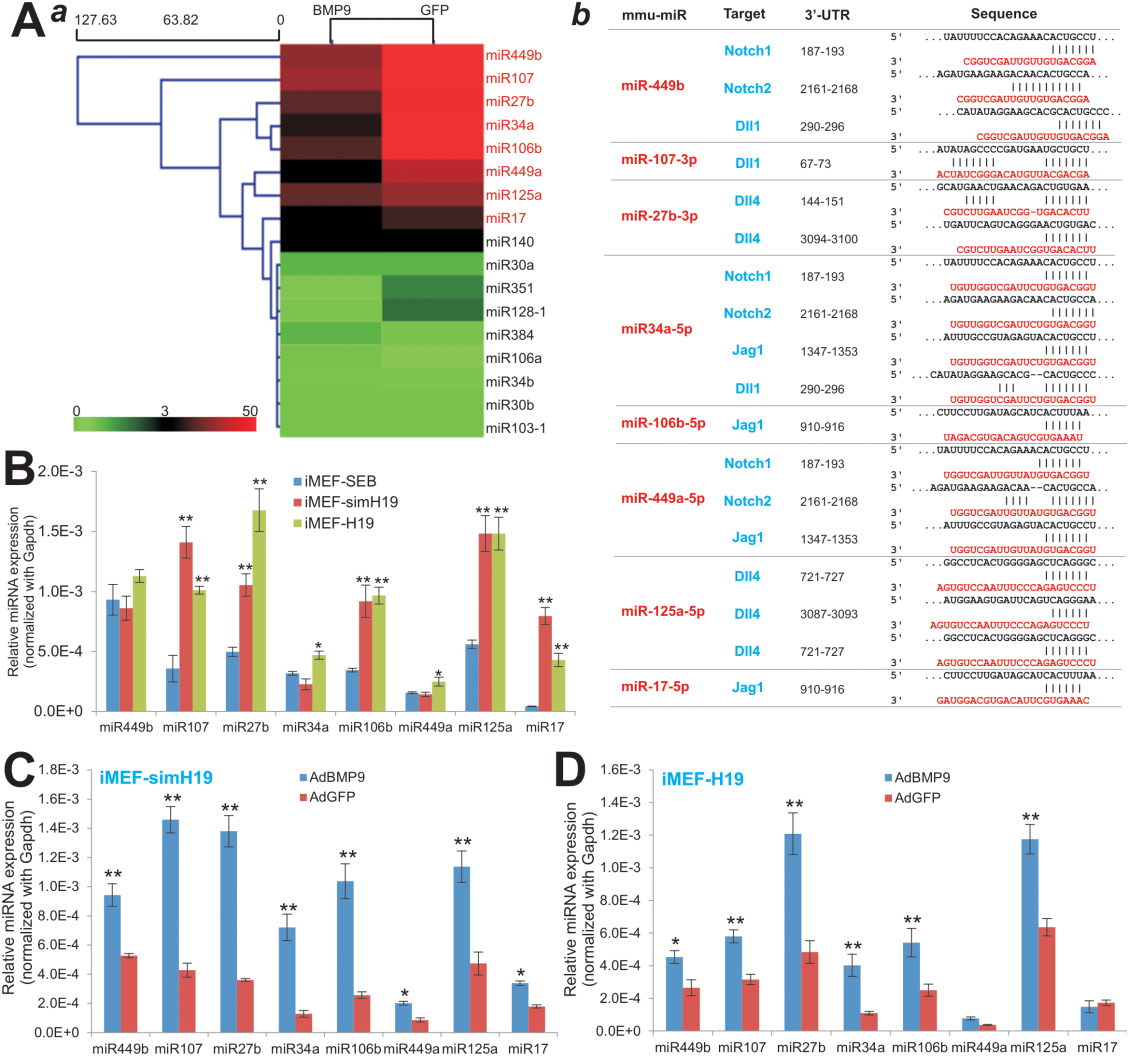
lncRNA H19 mediates BMP9-induced osteogenic differentiation by modulating the miRNAs that target Notch signaling **(A)** BMP9-regulated expression of Notch-targeting miRNAs in MSCs. Subconfluent iMEFs were infected with AdBMP9 or AdGFP for 36h. Total RNA was isolated from the infected iMEFs and subjected to TqPCR analysis of the selected 17 miRNAs that target the Notch pathway based on TargetScan. The relative normalized expression of these miRNAs was subjected to clustering analysis using the Multiple Experiment Viewer (MeV). The eight miRNAs that were significantly differentially expressed are highlighted in red (***a***). The Notch receptors/ligands and the putative target sites for the selected miRNAs are shown (***b***). **(B)** The basal expression levels of the eight miRNAs in iMEF-SEB, iMEF-simH19 and iMEF-H19 cells. Total RNA was isolated from the subconfluent cells and subjected to TqPCR. *Gapdh* was used as a reference gene. “*” *p < 0.05*, “**” *p < 0.001*, compared with that of the iMEF-SEB group. **(C)** The effect of BMP9 on the expression of miRNAs in iMEF-simH19 cells. The cells were infected with AdBMP9 or AdGFP for 36h. Total RNA was isolated and subjected to TqPCR analysis. *Gapdh* was used as a reference gene. “*” *p < 0.05*, “**” *p < 0.001*, compared with that of the AdGFP group. **(D)** The effect of BMP9 on the expression of miRNAs in iMEF-H19 cells. The cells were infected with AdBMP9 or AdGFP for 36h. Total RNA was isolated and subjected to TqPCR analysis. *Gapdh* was used as a reference gene. “*” *p<0.05*, “**” *p<0.001*, compared with that of the AdGFP group.

While the biological functions of lncRNAs remain to be fully understood, it has been shown that lncRNAs can act as precursors or sponges of micro-RNAs (miRNAs). We hypothesized that H19 may influence BMP9-induced osteogenic signaling by modulating miRNAs that target Notch receptors and/or ligands. Using the TargetScan program, we identified 17 miRNAs that were projected to target Notch receptors and ligands (Figure 8A–a). We first tested whether the expression of these miRNAs in MSCs were influenced by BMP9 stimulation. The qPCR results were subjected to clustering analysis using the MeV program (Figure 8A–a). The clustering analysis revealed that most of the selected miRNAs were down-regulated upon BMP9 stimulation, compared with that of the GFP control group, while nearly half of the selected miRNAs were expressed at very low levels in both BMP9 and GFP groups (Figure 8A–a). We found that eight miRNAs, including miR-449b, miR-107, miR-27b, miR-34a, miR-106b, miR-449a, miR-125a and miR-17, were most significantly differentially expressed upon BMP9 stimulation (Figure 8A–a). Interestingly, most of the eight miRNAs are predicted to target Notch ligands, especially Dll1, Dll4 and Jag1 (Figure 8A–b).

We next examined the expression status of the selected eight miRNAs in MSCs when H19 was silenced or constitutively expressed. When H19 was silenced, we found the expression of miR-107, miR-27b, miR-106b, miR125a and miR17 was significantly elevated while the expression of miR-449b, miR-34a and miR-449a was not significantly affected (Figure 8B). However, in the MSCs where H19 was constitutively expressed seven of the eight miRNAs were expressed at significantly higher levels, and only the expression of miR-449b did not change significantly (Figure 8B). These results suggest that deregulation of H19 expression may lead to the elevated expression of multiple miRNAs that target Notch receptors and/or ligands in MSCs.

To further study whether H19-regulated expression of these miRNAs was functionally associated with BMP9-induced osteogenic signaling, we infected the iMEF-simH19, iMEF-H19, and iMEF-SEB cells with AdBMP9 or AdGFP, and analyzed the expression profiles of the eight miRNAs. When H19 was silenced in MSCs, BMP9 stimulation led to a significantly elevated expression of all eight miRNAs, compared with that of the GFP group, although the increases in miR-449a and miR-17 were modest but significant (p<0.05) (Figure 8C). Furthermore, when the MSCs, in which H19 was constitutively expressed, were stimulated with BMP9, we found six of the eight miRNAs were significantly elevated, and only miR-449a and miR-17 did not show any significant changes in expression (Figure 8D). Thus, deregulated H19 expression led to the increased expression of at least six miRNAs (e.g., miR-449b, miR-107, miR-27b, miR-34a, miR-106b and miR-125a) in MSCs upon BMP9 stimulation. Interestingly, most of these upregulated miRNAs are projected to target the Notch ligands Dll1, Dll4 and Jag1, consistent with the findings that either silencing H19 or constitutive H19 expression leads to the decreased expression of Notch ligands in MSCs (Figure 7C). Taken together, these results strongly suggest that lncRNA H19 may modulate the expression of a group of miRNAs that can target Notch receptors and/or ligands, and may in turn participate in BMP9-induced osteogenic differentiation of MSCs.

## DISCUSSION

BMP9 was originally identified in developing mouse liver [28, 56]. Besides its osteogenic activity, BMP9 plays certain roles in inducing and maintaining embryonic basal forebrain cholinergic neurons, inhibiting hepatic glucose production and inducing the expression of key enzymes of lipid metabolism, stimulating hepcidin 1 expression, and regulating angiogenesis [28]. As BMP9 is one of the least studied BMP, we performed gene expression profiling analysis and identified several early downstream targets [28, 29, 57–62]. We also found BMP9 signaling has extensive cross-talks with other signaling pathways, especially Wnt and Notch signaling [29, 55, 63–69]. We found BMP9 up-regulates the Notch downstream target Hey1 [60], and that BMP9-promoted proliferation and tumor growth of osteosarcoma can be effectively blunted by blocking Notch signaling, suggesting that BMP9 may function as upstream of Notch signaling.

Notch signaling plays important roles in regulating cell fate decision, stem cell renewal and proliferation [20, 70]. The Notch pathway includes Notch receptors (Notch 1-4), ligands (DLLs and JAGs), negative and positive regulators, and transcription factors [20]. Upon ligand binding, Notch proteins are proteolytically cleaved in two steps by ADAM10 and γ-secretase, resulting in the Notch intracellular domain (NICD) which is translocated to the nucleus, interacting with transcription factor CSL/RBPJk and the mastermind-like protein, and regulating CSL target genes, such as the HES family and Hes-related repressor genes [20, 70]. Notch signaling plays a critical role in bone and skeletal development as Notch knockout mice exhibit severe skeletal abnormalities [18, 71, 72]. DLL3 Mutations cause spondylocostal dysostosis in humans [70, 72]. A forced expression of Notch ligands and receptors impairs both osteoblastic and osteoclastic differentiation of the progenitor cells [18, 70, 72]. Interestingly, we recently demonstrated that a blockade of Notch signaling significantly impairs BMP9-induced osteogenic differentiation and angiogenesis [73]. Nonetheless, it remains to be fully elucidated about how BMP9 regulates Notch signaling during osteogenesis.

In this study, we demonstrate that lncRNA H19 may play an important role in mediating BMP9-induced osteogenic signaling in MSCs. Although H19 was identified nearly three decades ago [43, 74], the diverse biological functions of H19 remain to be fully understood [44–47]. H19 plays important roles in embryonal development and growth control [75], and is located in a highly conserved imprinted gene cluster, which also contains a neighboring reciprocally imprinted gene for insulin-like growth factor 2 (*Igf2*). This cluster is coordinately regulated by the differentially methylated regions (DMR), an imprinting control region (ICR), and the common endodermal and mesodermal enhancers [44, 75]. While *Igf2* is paternally expressed, both human and mouse *H19* is expressed from the maternal allele. *H19* expression is strongly induced during embryogenesis and downregulated after birth, except in adult skeletal muscle and heart [44, 75]. Interestingly, mice with targeted deletions of H19, H19^Δ13^ (deleted the gene and 10 kb upstream, including the ICR) and H19^Δ3^ (deleted only the 3-kb transcription unit) were viable without apparent phenotype, except for an increase in placental and fetal weight, with a newborn weight of 27% for H19^Δ13^ and 8% for H19^Δ3^ [76, 77], which was later linked to loss of imprinting on the maternal allele and biallelic expression of *Igf2*, revealing a cis effect of these targeted deletions on Igf2 expression [44, 75, 78]. Furthermore, conflicting results were reported about the role of H19 as a tumor suppressor or oncogene [45, 79–83]. These seemingly conflicting findings reflect the complex co-regulatory circuitry of *H19-Igf2* expression in the highly conserved imprinted gene cluster [84]. In fact, it has been reported that the H19 antisense lncRNA 91H may play an important role in modulating *H19-Igf2* expression although the exact mechanism remains to be fully understood [85–87]. Thus, it is important to further investigate if lncRNA 91H is regulated by BMP9 and/or whether lncRNA 91H regulates H19 functions in BMP9-induced osteogenic differentiation of MSCs.

While functioning as a noncoding RNA itself, H19 has been recently shown to encode miR-675, which is mainly detected in the mouse placenta and at a time window during which placental growth normally ceases [88]. Interestingly, it was reported that H19-derived miR-675 promoted osteoblast differentiation through TGFβ/Smad/HDAC pathway [89]. However, we did not observed any expression correlations between H19 and miR-675 at different time points in the MSCs stimulated with BMP9 (data not shown). While the exact biological functions of H19 remain enigmatic, one aspect of H19 actions may regulate miRNA functions by serving as a molecular sponge. In fact, it has been recently demonstrated that H19 modulates let-7 availability by acting as a molecular sponge [90]. It was reported that overexpression of H19 and miR-675 inhibited adipogenesis, possibly due to the fact that miR-675 targeted the 3′ untranslated regions of the histone deacetylase (HDAC) 4-6 transcripts and resulted in deregulation of HDACs 4-6 [91]. On the other hand, H19 was reported to activate Wnt signaling and promote osteoblast differentiation by acting as a competing endogenous RNA (or ceRNA) [92].

In this study, we investigate whether H19 modulates the expression levels of a panel of miRNAs that are predicted to modulate Notch ligands and receptors upon BMP9 stimulation in MSCs. Our results strongly suggest that Notch-associated miRNAs (e.g., miR-107, miR-27b, miR-106b, miR125a and miR17) may be modulated by H19 in response to BMP9 stimulation in MSCs although the detailed mechanisms remain to be understood. The paradoxical effects of constitutive H19 expression and silencing H19 expression on the expression of the above miRNAs may reflect the complex regulatory circuitry of H19 expression, but need to be fully investigated. Furthermore, it remains to be elucidated how BMP9 signaling regulates H19 expression in MSCs, given the complexity of the imprinted *H19/Igf2* cluster. Nonetheless, our findings demonstrate that H19 plays an important role in mediating BMP9-induced osteogenic differentiation of MSCs.

## MATERIALS AND METHODS

### Cell culture and chemicals

Mouse mesenchymal stem cell (MSC) lines iMEFs and iMADs are the conditionally immortalized mouse embryonic fibroblasts and immortalized multipotent adipose-derived cells as previously described [48–50]. 293pTP cells were derived from HEK-293 cells overexpressing human Ad5 pTP gene as previously characterized [93]. All above cell lines were maintained in Dulbecco’s Modified Eagle Medium (DMEM) supplemented with 10% fetal bovine serum (FBS, Gemini Bio-Products, West Sacramento, CA), containing 100 U/ml penicillin and 100 mg/ml streptomycin, at 37°C in 5% CO_2_ as described [94–96]. Unless indicated otherwise, all other reagents were purchased from Sigma-Aldrich (St. Louis, MO) or Thermo Fisher Scientific (Waltham, MA).

### Construction and generation of recombinant adenoviral vectors AdBMP9, AdNICD1, AdH19, and AdsimH19

Recombinant adenoviruses were generated using the AdEasy technology as described [97, 98]. The AdBMP9 was previously characterized [26, 27, 52, 55, 99]. Briefly, the coding region of human BMP9 and the intracellular domain (NICD1) of human NOTCH1, and the full-length transcript of mouse lncRNA H19 were PCR amplified and subcloned into an adenoviral shuttle vector, and used to generate recombinant adenoviral vectors, resulting in pAd5-BMP9, pAd5-NICD1 and pAd5-H19, which were subsequently used to generate recombinant adenoviruses in HEK-293 or 293pTP cells. AdBMP9 also co-expresses enhanced GFP (eGFP), while AdNICD1 and AdH19 co-express monomeric RFP (mRFP). AdGFP was used as a mock virus control [48, 63]. For making AdsimH19, three siRNAs targeting mouse H19 were simultaneously assembled to an adenoviral shuttle vector using the Gibson Assembly system as described [100, 101]. For all adenoviral infections, polybrene (4-8μg/ml) was added to enhance infection efficiency as previously described [102]. Detailed information about vector constructions is available upon request.

### Retrovirus transduction and establishment of MSC lines stably expressing mouse H19 and simH19

To express full-length mouse lncRNA H19, the full-length transcript of mouse H19 was PCR amplified and cloned to the homemade retroviral vector pSEB-flag which is driven by the hEFH promoter, resulting in pSEB-H19. For silencing mouse H19, three siRNAs targeting mouse H19 were designed by the *siDESIGN* program and subcloned into the homemade retroviral vector pSOK which contains the converging Pol III promoters U6 and H1 to drive the expression of siRNAs [100, 101]. All constructs were verified by DNA sequencing. Both retroviral vectors confer blasticidin resistance.

To package retroviruses, the retroviral vectors or the empty control vector were co-transfected with the packaging plasmid pCL-AMPHO into HEK-293 cells. The packaged retrovirus supernatants were collected at 36h, 48h, 60h and 72h respectively, and used to infect subconfluent iMEFs. At 24h post infection, the infected iMEFs were selected in blasticidin (4μg/ml) for 5 to 7 days [103]. The resultant stable iMEF lines were designated as iMEF-SEB, iMEF-simH19, and iMEF-H19, respectively.

### RNA isolation and touchdown quantitative Real-Time PCR (TqPCR)

Total RNA was isolated with TRIZOL Reagent (Invitrogen, Carlsbad, CA) according to the manufacturer’s instructions and subjected to reverse transcription reactions using hexamer and M-MuLV Reverse Transcriptase (New England Biolabs, Ipswich, MA). The resultant cDNA products were diluted 10- to 100-fold and used as PCR templates. PCR primers were designed by using the Primer3 Plus program [104]. The quantitative PCR analysis was carried out using our recently optimized TqPCR protocol [51, 105]. Briefly, the SYBR Green qPCR reactions (Bimake, Houston, TX) were set up according to manufacturer’s instructions. The cycling program was modified by incorporating 4 cycles of touchdown steps prior to the regular cycling program as described [51, 105]. *Gapdh* was used as a reference gene. All sample values were normalized to *Gapdh* expression by using the 2^−ΔΔCt^ method. The qPCR primer sequences are listed in Supplementary Table 1.

### Alkaline phosphatase (ALP) assays

The ALP activities were assessed quantitatively and qualitatively using the modified Great Escape SEAP chemiluminescence assay (BD Clontech) and/or histochemical staining as described previously [64, 106]. Briefly, for the histochemical staining, the cells were fixed with 0.05% glutaradehyde at room temperature for 10 min. After being washed with PBS, cells were stained subjected to histochemical staining with a mixture of 0.1 mg/mL of napthol AS-MX phosphate and 0.6 mg/mL of Fast Blue BB salt. After 20 minutes, the mixture was removed and replaced with PBS. Histochemical staining was recorded using bright light microscopy. For the chemiluminescence assay, the cells were lysed by the Cell Culture Lysis Buffer (Promega, Madison, WI). Then 5μl Cell Lysis Buffer, 5ul substrate (BD Clontech) and 15μl Lupo Buffer were mixed well under a light-proof condition, and incubated at room temperature for 20 minutes before measuring chemiluminescence signals. Each assay condition was performed in triplicate. The results were repeated in at least three independent batches of experiments. ALP activities were normalized by total cellular protein concentrations among the samples.

### Matrix mineralization assay (Alizarin red S staining)

MSC cells were seeded in 24-well cell culture plates and infected with the indicated adenoviruses. Infected cells were cultured in the presence of ascorbic acid (50 mg/ml) and β-glycerophosphate (10mM). At the desired time points, mineralized matrix nodules were stained for calcium precipitation by means of Alizarin Red S staining as described [26, 68]. Briefly, cells were fixed with 1% glutaraldehyde for 10 min. After washing with PBS, cells were incubated with 2% Alizarin Red S at room temperature for 30 min, followed by washing with acidic PBS (pH=4.2). The staining of calcium mineral deposits were recorded under bright field microscopy.

### Subcutaneous stem cell implantation and ectopic bone formation

The use and care of animals in this study were approved by the Institutional Animal Care and Use Committee. All experimental procedures were carried out in accordance with the approved guidelines. Subcutaneous stem cell implantation procedure was performed as described [27, 48, 101, 107]. Briefly, the iMEF-SEB, iMEF-simH19 and iMEF-H19 cells were infected with different adenoviruses. After 24h cells were collected and resuspended in PBS (80ul each injection). The cells were injected subcutaneously into the flanks of athymic nude mice (Envigo/Harlan Research Laboratories; n=5/group, female, 5–6 week old; 2 × 10^6^ cells per injection site). The animals were maintained ad lib in the biosafety barrier facility. At the endpoints, animals were euthanized, and the ectopic masses were retrieved from injection sites and subjected to μCT imaging, followed by histological and other specialty staining evaluations.

### Micro-computed tomographic (μCT) imaging analysis

The retrieved samples were fixed in 10% formalin and imaged with the micro-CT (μCT) component of the GE triumph (GE Healthcare) trimodality preclinical imaging system. All image data were analyzed by Amira 5.3 (Visage Imaging, Inc.), and 3D volumetric data and bone density were determined as previously described [65, 108, 109].

### Hematoxylin and eosin (H & E) and Masson's trichrome staining

The retrieved specimens were fixed with 10% formalin, decalcified and embedded in paraffin. Serial sections at 5μm of the embedded specimens were carried out, and mounted onto treated slides. Then the sections were deparaffinized and then rehydrated in a graduated fashion. H & E staining and Masson’s trichrome staining were done as described [68, 110, 111].

### Western blot analysis

Western blot analysis was done as described [112–114]. The cell lysates were prepared using cell lysis buffer containing a protease inhibitor PMSF. About 50 mg of total protein for each sample was loaded into 10% SDS-PAGE gel and transferred to PVDF membranes, which were blocked and incubated overnight with primary antibodies against NICD1 (Santa Cruz Biotechnology, Santa Cruz, CA) and GAPDH (Santa Cruz Biotechnology) at a dilution of 1:200 and 1:1000, respectively. After being washed, the membranes were incubated with a secondary antibody conjugated with horseradish peroxidase. Immune-reactive signals were detected using ECL kit (Millipore, America). Image J software were used to quantitatively analysis.

### Immunofluorescence staining

Subconfluent cells were treated with drug and/or adenoviral infection and fixed with 4% paraformaldehyde. The fixed cells were then treated with 0.1% Triton-100 and blocked with 10% bovine serum albumin. Cells were incubated with Notch1 antibody (against the NICD domain) (Santa Cruz Biotechnology) in 4°C overnight and stained with Cy3-anti-mouse IgG secondary antibody (Jackson ImmunoResearch). Cell nuclei were counter-stained with DAPI, followed by fluorescence microscopic imaging.

### Statistical analysis

All quantitative experiments were performed in triplicate and/or repeated in three independent batches of experiments. Data were expressed as mean ± standard deviation (SD). The one-way analysis of variance was used to analyze statistical significance. A value of *p<0.05* was considered statistically significant.

## SUPPLEMENTARY TABLE



## References

[1] Prockop DJ (1997). Marrow stromal cells as stem cells for nonhematopoietic tissues. Science.

[2] Caplan AI, Bruder SP (2001). Mesenchymal stem cells: building blocks for molecular medicine in the 21st century. Trends Mol Med.

[3] Deng ZL, Sharff KA, Tang N, Song WX, Luo J, Luo X, Chen J, Bennett E, Reid R, Manning D, Xue A, Montag AG, Luu HH (2008). Regulation of osteogenic differentiation during skeletal development. Front Biosci.

[4] Rastegar F, Shenaq D, Huang J, Zhang W, Zhang BQ, He BC, Chen L, Zuo GW, Luo Q, Shi Q, Wagner ER, Huang E, Gao Y (2010). Mesenchymal stem cells: Molecular characteristics and clinical applications. World J Stem Cells.

[5] Shenaq DS, Rastegar F, Petkovic D, Zhang BQ, He BC, Chen L, Zuo GW, Luo Q, Shi Q, Wagner ER, Huang E, Gao Y, Gao JL (2010). Mesenchymal Progenitor Cells and Their Orthopedic Applications: Forging a Path towards Clinical Trials. Stem Cells Int.

[6] Teven CM, Liu X, Hu N, Tang N, Kim SH, Huang E, Yang K, Li M, Gao JL, Liu H, Natale RB, Luther G, Luo Q (2011). Epigenetic regulation of mesenchymal stem cells: a focus on osteogenic and adipogenic differentiation. Stem Cells Int.

[7] Noel D, Djouad F, Jorgense C (2002). Regenerative medicine through mesenchymal stem cells for bone and cartilage repair. Curr Opin Investig Drugs.

[8] Chan JL, Tang KC, Patel AP, Bonilla LM, Pierobon N, Ponzio NM, Rameshwar P (2006). Antigen-presenting property of mesenchymal stem cells occurs during a narrow window at low levels of interferon-gamma. Blood.

[9] Corcione A, Benvenuto F, Ferretti E, Giunti D, Cappiello V, Cazzanti F, Risso M, Gualandi F, Mancardi GL, Pistoia V, Uccelli A (2006). Human mesenchymal stem cells modulate B-cell functions. Blood.

[10] Djouad F, Charbonnier LM, Bouffi C, Louis-Plence P, Bony C, Apparailly F, Cantos C, Jorgensen C, Noel D (2007). Mesenchymal stem cells inhibit the differentiation of dendritic cells through an interleukin-6-dependent mechanism. Stem Cells.

[11] Olsen BR, Reginato AM, Wang W (2000). Bone development. Annu Rev Cell Dev Biol.

[12] Raucci A, Bellosta P, Grassi R, Basilico C, Mansukhani A (2008). Osteoblast proliferation or differentiation is regulated by relative strengths of opposing signaling pathways. J Cell Physiol.

[13] Kim JH, Liu X, Wang J, Chen X, Zhang H, Kim SH, Cui J, Li R, Zhang W, Kong Y, Zhang J, Shui W, Lamplot J (2013). Wnt signaling in bone formation and its therapeutic potential for bone diseases. Ther Adv Musculoskelet Dis.

[14] Yang K, Wang X, Zhang H, Wang Z, Nan G, Li Y, Zhang F, Mohammed MK, Haydon RC, Luu HH, Bi Y, He TC (2016). The evolving roles of canonical WNT signaling in stem cells and tumorigenesis: implications in targeted cancer therapies. Lab Invest.

[15] Denduluri SK, Olumuyiwa Idowu O, Wang Z, Liao Z, Yan Z, Mohammed MK, Ye J, Wei Q, Wang J, Zhao L, Luu HH (2015). Insulin-like growth factor (IGF) signaling in tumorigenesis and the development of cancer drug resistance. Genes Dis.

[16] Teven CM, Farina EM, Rivas J, Reid RR (2014). Fibroblast growth factor (FGF) signaling in development and skeletal diseases. Genes Dis.

[17] Jo A, Denduluri SK, Zhang B, Wang Z, Yin L, Yan Z, Kang R, Shi LL, Mok J, Lee MJ, Haydon RC (2014). The Versatile Functions of Sox9 in Development, Stem Cells, and Human Diseases. Genes Dis.

[18] Louvi A, Artavanis-Tsakonas S (2012). Notch and disease: a growing field. Semin Cell Dev Biol.

[19] Zanotti S, Canalis E (2010). Notch and the skeleton. Mol Cell Biol.

[20] Guruharsha KG, Kankel MW, Artavanis-Tsakonas S (2012). The Notch signalling system: recent insights into the complexity of a conserved pathway. Nat Rev Genet.

[21] Zhang F, Song J, Zhang H, Huang E, Song D, Tollemar V, Wang J, Wang J, Mohammed M, Wei Q, Fan J, Liao L, Zou Y (2016). Wnt and BMP signaling crosstalk in regulating dental stem cells: Implications in dental tissue engineering. Genes & Diseases.

[22] Luu HH, Song WX, Luo X, Manning D, Luo J, Deng ZL, Sharff KA, Montag AG, Haydon RC, He TC (2007). Distinct roles of bone morphogenetic proteins in osteogenic differentiation of mesenchymal stem cells. J Orthop Res.

[23] Wang RN, Green J, Wang Z, Deng Y, Qiao M, Peabody M, Zhang Q, Ye J, Yan Z, Denduluri S, Idowu O, Li M, Shen C (2014). Bone Morphogenetic Protein (BMP) signaling in development and human diseases. Genes Dis.

[24] Reddi AH (1998). Role of morphogenetic proteins in skeletal tissue engineering and regeneration. Nat Biotechnol.

[25] Zou H, Choe KM, Lu Y, Massague J, Niswander L (1997). BMP signaling and vertebrate limb development. Cold Spring Harb Symp Quant Biol.

[26] Cheng H, Jiang W, Phillips FM, Haydon RC, Peng Y, Zhou L, Luu HH, An N, Breyer B, Vanichakarn P, Szatkowski JP, Park JY, He TC (2003). Osteogenic activity of the fourteen types of human bone morphogenetic proteins (BMPs). J Bone Joint Surg Am.

[27] Kang Q, Sun MH, Cheng H, Peng Y, Montag AG, Deyrup AT, Jiang W, Luu HH, Luo J, Szatkowski JP, Vanichakarn P, Park JY, Li Y (2004). Characterization of the distinct orthotopic bone-forming activity of 14 BMPs using recombinant adenovirus-mediated gene delivery. Gene Ther.

[28] Luther G, Wagner ER, Zhu G, Kang Q, Luo Q, Lamplot J, Bi Y, Luo X, Luo J, Teven C, Shi Q, Kim SH, Gao JL (2011). BMP-9 Induced Osteogenic Differentiation of Mesenchymal Stem Cells: Molecular Mechanism and Therapeutic Potential. Curr Gene Ther.

[29] Lamplot JD, Qin J, Nan G, Wang J, Liu X, Yin L, Tomal J, Li R, Shui W, Zhang H, Kim SH, Zhang W, Zhang J (2013). BMP9 signaling in stem cell differentiation and osteogenesis. Am J Stem Cells.

[30] Wang Y, Hong S, Li M, Zhang J, Bi Y, He Y, Liu X, Nan G, Su Y, Zhu G, Li R, Zhang W, Wang J (2013). Noggin resistance contributes to the potent osteogenic capability of BMP9 in mesenchymal stem cells. J Orthop Res.

[31] Luo J, Tang M, Huang J, He BC, Gao JL, Chen L, Zuo GW, Zhang W, Luo Q, Shi Q, Zhang BQ, Bi Y, Luo X (2010). TGFbeta/BMP type I receptors ALK1 and ALK2 are essential for BMP9-induced osteogenic signaling in mesenchymal stem cells. J Biol Chem.

[32] Guttman M, Amit I, Garber M, French C, Lin MF, Feldser D, Huarte M, Zuk O, Carey BW, Cassady JP, Cabili MN, Jaenisch R, Mikkelsen TS (2009). Chromatin signature reveals over a thousand highly conserved large non-coding RNAs in mammals. Nature.

[33] Carninci P, Kasukawa T, Katayama S, Gough J, Frith MC, Maeda N, Oyama R, Ravasi T, Lenhard B, Wells C, Kodzius R, Shimokawa K, Bajic VB (2005). The transcriptional landscape of the mammalian genome. Science.

[34] Brosnan CA, Voinnet O (2009). The long and the short of noncoding RNAs. Curr Opin Cell Biol.

[35] Jacquier A (2009). The complex eukaryotic transcriptome: unexpected pervasive transcription and novel small RNAs. Nat Rev Genet.

[36] Berretta J, Morillon A (2009). Pervasive transcription constitutes a new level of eukaryotic genome regulation. EMBO Rep.

[37] Djebali S, Davis CA, Merkel A, Dobin A, Lassmann T, Mortazavi A, Tanzer A, Lagarde J, Lin W, Schlesinger F, Xue C, Marinov GK, Khatun J (2012). Landscape of transcription in human cells. Nature.

[38] Quinn JJ, Chang HY (2015). Unique features of long non-coding RNA biogenesis and function. Nat Rev Genet.

[39] Quinodoz S, Guttman M (2014). Long noncoding RNAs: an emerging link between gene regulation and nuclear organization. Trends Cell Biol.

[40] Johnsson P, Lipovich L, Grander D, Morris KV (2014). Evolutionary conservation of long non-coding RNAs; sequence, structure, function. Biochim Biophys Acta.

[41] Hung T, Chang HY (2010). Long noncoding RNA in genome regulation: prospects and mechanisms. RNA Biol.

[42] Mattick JS, Makunin IV (2006). Non-coding RNA. Hum Mol Genet.

[43] Bartolomei MS, Zemel S, Tilghman SM (1991). Parental imprinting of the mouse H19 gene. Nature.

[44] Raveh E, Matouk IJ, Gilon M, Hochberg A (2015). The H19 Long non-coding RNA in cancer initiation, progression and metastasis - a proposed unifying theory. Mol Cancer.

[45] Matouk IJ, Halle D, Raveh E, Gilon M, Sorin V, Hochberg A (2016). The role of the oncofetal H19 lncRNA in tumor metastasis: orchestrating the EMT-MET decision. Oncotarget.

[46] Venkatraman A, He XC, Thorvaldsen JL, Sugimura R, Perry JM, Tao F, Zhao M, Christenson MK, Sanchez R, Yu JY, Peng L, Haug JS, Paulson A (2013). Maternal imprinting at the H19-Igf2 locus maintains adult haematopoietic stem cell quiescence. Nature.

[47] Goodell MA (2013). Parental permissions: H19 and keeping the stem cell progeny under control. Cell Stem Cell.

[48] Huang E, Bi Y, Jiang W, Luo X, Yang K, Gao JL, Gao Y, Luo Q, Shi Q, Kim SH, Liu X, Li M, Hu N (2012). Conditionally Immortalized Mouse Embryonic Fibroblasts Retain Proliferative Activity without Compromising Multipotent Differentiation Potential. PLoS One.

[49] Wang N, Zhang W, Cui J, Zhang H, Chen X, Li R, Wu N, Chen X, Wen S, Zhang J, Yin L, Deng F, Liao Z (2014). The piggyBac Transposon-Mediated Expression of SV40 T Antigen Efficiently Immortalizes Mouse Embryonic Fibroblasts (MEFs). PLoS One.

[50] Lu S, Wang J, Ye J, Zou Y, Zhu Y, Wei Q, Wang X, Tang S, Liu H, Fan J, Zhang F, Farina EM, Mohammed MM (2016). Bone morphogenetic protein 9 (BMP9) induces effective bone formation from reversibly immortalized multipotent adipose-derived (iMAD) mesenchymal stem cells. Am J Transl Res.

[51] Zhang Q, Wang J, Deng F, Yan Z, Xia Y, Wang Z, Ye J, Deng Y, Zhang Z, Qiao M, Li R, Denduluri SK, Wei Q (2015). TqPCR: A Touchdown qPCR Assay with Significantly Improved Detection Sensitivity and Amplification Efficiency of SYBR Green qPCR. PLoS One.

[52] Kang Q, Song WX, Luo Q, Tang N, Luo J, Luo X, Chen J, Bi Y, He BC, Park JK, Jiang W, Tang Y, Huang J (2009). A comprehensive analysis of the dual roles of BMPs in regulating adipogenic and osteogenic differentiation of mesenchymal progenitor cells. Stem Cells Dev.

[53] Canalis E (2008). Notch signaling in osteoblasts. Sci Signal.

[54] Engin F, Lee B (2010). NOTCHing the bone: insights into multi-functionality. Bone.

[55] Li R, Zhang W, Cui J, Shui W, Yin L, Wang Y, Zhang H, Wang N, Wu N, Nan G, Chen X, Wen S, Deng F (2014). Targeting BMP9-promoted human osteosarcoma growth by inactivation of notch signaling. Curr Cancer Drug Targets.

[56] Song JJ, Celeste AJ, Kong FM, Jirtle RL, Rosen V, Thies RS (1995). Bone morphogenetic protein-9 binds to liver cells and stimulates proliferation. Endocrinology.

[57] Peng Y, Kang Q, Cheng H, Li X, Sun MH, Jiang W, Luu HH, Park JY, Haydon RC, He TC (2003). Transcriptional characterization of bone morphogenetic proteins (BMPs)-mediated osteogenic signaling. J Cell Biochem.

[58] Peng Y, Kang Q, Luo Q, Jiang W, Si W, Liu BA, Luu HH, Park JK, Li X, Luo J, Montag AG, Haydon RC, He TC (2004). Inhibitor of DNA binding/differentiation helix-loop-helix proteins mediate bone morphogenetic protein-induced osteoblast differentiation of mesenchymal stem cells. J Biol Chem.

[59] Luo Q, Kang Q, Si W, Jiang W, Park JK, Peng Y, Li X, Luu HH, Luo J, Montag AG, Haydon RC, He TC (2004). Connective Tissue Growth Factor (CTGF) Is Regulated by Wnt and Bone Morphogenetic Proteins Signaling in Osteoblast Differentiation of Mesenchymal Stem Cells. J Biol Chem.

[60] Sharff KA, Song WX, Luo X, Tang N, Luo J, Chen J, Bi Y, He BC, Huang J, Li X, Jiang W, Zhu GH, Su Y (2009). Hey1 Basic Helix-Loop-Helix Protein Plays an Important Role in Mediating BMP9-induced Osteogenic Differentiation of Mesenchymal Progenitor Cells. J Biol Chem.

[61] Huang E, Zhu G, Jiang W, Yang K, Gao Y, Luo Q, Gao JL, Kim SH, Liu X, Li M, Shi Q, Hu N, Wang L (2012). Growth hormone synergizes with BMP9 in osteogenic differentiation by activating the JAK/STAT/IGF1 pathway in murine multilineage cells. J Bone Miner Res.

[62] Zhang J, Weng Y, Liu X, Wang J, Zhang W, Kim SH, Zhang H, Li R, Kong Y, Chen X, Shui W, Wang N, Zhao C (2013). Endoplasmic reticulum (ER) stress inducible factor cysteine-rich with EGF-like domains 2 (Creld2) is an important mediator of BMP9-regulated osteogenic differentiation of mesenchymal stem cells. PLoS One.

[63] Tang N, Song WX, Luo J, Luo X, Chen J, Sharff KA, Bi Y, He BC, Huang JY, Zhu GH, Su YX, Jiang W, Tang M (2009). BMP9-induced osteogenic differentiation of mesenchymal progenitors requires functional canonical Wnt/beta-catenin signaling. J Cell Mol Med.

[64] Zhang W, Deng ZL, Chen L, Zuo GW, Luo Q, Shi Q, Zhang BQ, Wagner ER, Rastegar F, Kim SH, Jiang W, Shen J, Huang E (2010). Retinoic acids potentiate BMP9-induced osteogenic differentiation of mesenchymal progenitor cells. PLoS One.

[65] Chen L, Jiang W, Huang J, He BC, Zuo GW, Zhang W, Luo Q, Shi Q, Zhang BQ, Wagner ER, Luo J, Tang M, Wietholt C (2010). Insulin-like growth factor 2 (IGF-2) potentiates BMP-9-induced osteogenic differentiation and bone formation. J Bone Miner Res.

[66] Liu X, Qin J, Luo Q, Bi Y, Zhu G, Jiang W, Kim SH, Li M, Su Y, Nan G, Cui J, Zhang W, Li R (2013). Cross-talk between EGF and BMP9 signalling pathways regulates the osteogenic differentiation of mesenchymal stem cells. J Cell Mol Med.

[67] Hu N, Jiang D, Huang E, Liu X, Li R, Liang X, Kim SH, Chen X, Gao JL, Zhang H, Zhang W, Kong YH, Zhang J (2013). BMP9-regulated angiogenic signaling plays an important role in the osteogenic differentiation of mesenchymal progenitor cells. J Cell Sci.

[68] Zhang H, Wang J, Deng F, Huang E, Yan Z, Wang Z, Deng Y, Zhang Q, Zhang Z, Ye J, Qiao M, Li R, Wang J (2015). Canonical Wnt signaling acts synergistically on BMP9-induced osteo/odontoblastic differentiation of stem cells of dental apical papilla (SCAPs). Biomaterials.

[69] Zhang H, Li L, Dong Q, Wang Y, Feng Q, Ou X, Zhou P, He T, Luo J (2015). Activation of PKA/CREB Signaling is Involved in BMP9-Induced Osteogenic Differentiation of Mesenchymal Stem Cells. Cell Physiol Biochem.

[70] Penton AL, Leonard LD, Spinner NB (2012). Notch signaling in human development and disease. Semin Cell Dev Biol.

[71] Andersson ER, Sandberg R, Lendahl U (2011). Notch signaling: simplicity in design, versatility in function. Development.

[72] Tao J, Chen S, Lee B (2010). Alteration of Notch signaling in skeletal development and disease. Ann N Y Acad Sci.

[73] Liao J, Wei Q, Zou Y, Fan J, Song D, Cui J, Zhang W, Zhu Y, Ma C, Hu X, Qu X, Chen L, Yu X (2017). Notch Signaling Augments BMP9-Induced Bone Formation by Promoting the Osteogenesis-Angiogenesis Coupling Process in Mesenchymal Stem Cells (MSCs). Cell Physiol Biochem.

[74] Brannan CI, Dees EC, Ingram RS, Tilghman SM (1990). The product of the H19 gene may function as an RNA. Mol Cell Biol.

[75] Gabory A, Jammes H, Dandolo L (2010). The H19 locus: role of an imprinted non-coding RNA in growth and development. Bioessays.

[76] Leighton PA, Ingram RS, Eggenschwiler J, Efstratiadis A, Tilghman SM (1995). Disruption of imprinting caused by deletion of the H19 gene region in mice. Nature.

[77] Ripoche MA, Kress C, Poirier F, Dandolo L (1997). Deletion of the H19 transcription unit reveals the existence of a putative imprinting control element. Genes Dev.

[78] Leighton PA, Saam JR, Ingram RS, Stewart CL, Tilghman SM (1995). An enhancer deletion affects both H19 and Igf2 expression. Genes Dev.

[79] Hao Y, Crenshaw T, Moulton T, Newcomb E, Tycko B (1993). Tumour-suppressor activity of H19 RNA. Nature.

[80] Lustig-Yariv O, Schulze E, Komitowski D, Erdmann V, Schneider T, de Groot N, Hochberg A (1997). The expression of the imprinted genes H19 and IGF-2 in choriocarcinoma cell lines. Is H19 a tumor suppressor gene?. Oncogene.

[81] Matouk IJ, DeGroot N, Mezan S, Ayesh S, Abu-lail R, Hochberg A, Galun E (2007). The H19 non-coding RNA is essential for human tumor growth. PLoS One.

[82] Yoshimizu T, Miroglio A, Ripoche MA, Gabory A, Vernucci M, Riccio A, Colnot S, Godard C, Terris B, Jammes H, Dandolo L (2008). The H19 locus acts *in vivo* as a tumor suppressor. Proc Natl Acad Sci U S A.

[83] Cui H, Hedborg F, He L, Nordenskjold A, Sandstedt B, Pfeifer-Ohlsson S, Ohlsson R (1997). Inactivation of H19, an imprinted and putative tumor repressor gene, is a preneoplastic event during Wilms’ tumorigenesis. Cancer Res.

[84] Tabano S, Colapietro P, Cetin I, Grati FR, Zanutto S, Mando C, Antonazzo P, Pileri P, Rossella F, Larizza L, Sirchia SM, Miozzo M (2010). Epigenetic modulation of the IGF2/H19 imprinted domain in human embryonic and extra-embryonic compartments and its possible role in fetal growth restriction. Epigenetics.

[85] Berteaux N, Aptel N, Cathala G, Genton C, Coll J, Daccache A, Spruyt N, Hondermarck H, Dugimont T, Curgy JJ, Forne T, Adriaenssens E (2008). A novel H19 antisense RNA overexpressed in breast cancer contributes to paternal IGF2 expression. Mol Cell Biol.

[86] Tran VG, Court F, Duputie A, Antoine E, Aptel N, Milligan L, Carbonell F, Lelay-Taha MN, Piette J, Weber M, Montarras D, Pinset C, Dandolo L (2012). H19 antisense RNA can up-regulate Igf2 transcription by activation of a novel promoter in mouse myoblasts. PLoS One.

[87] Gao T, He B, Pan Y, Xu Y, Li R, Deng Q, Sun H, Wang S (2015). Long non-coding RNA 91H contributes to the occurrence and progression of esophageal squamous cell carcinoma by inhibiting IGF2 expression. Mol Carcinog.

[88] Keniry A, Oxley D, Monnier P, Kyba M, Dandolo L, Smits G, Reik W (2012). The H19 lincRNA is a developmental reservoir of miR-675 that suppresses growth and Igf1r. Nat Cell Biol.

[89] Huang Y, Zheng Y, Jia L, Li W (2015). Long Noncoding RNA H19 Promotes Osteoblast Differentiation Via TGF-beta1/Smad3/HDAC Signaling Pathway by Deriving miR-675. Stem Cells.

[90] Kallen AN, Zhou XB, Xu J, Qiao C, Ma J, Yan L, Lu L, Liu C, Yi JS, Zhang H, Min W, Bennett AM, Gregory RI (2013). The imprinted H19 lncRNA antagonizes let-7 microRNAs. Mol Cell.

[91] Huang Y, Zheng Y, Jin C, Li X, Jia L, Li W (2016). Long Non-coding RNA H19 Inhibits Adipocyte Differentiation of Bone Marrow Mesenchymal Stem Cells through Epigenetic Modulation of Histone Deacetylases. Sci Rep.

[92] Liang WC, Fu WM, Wang YB, Sun YX, Xu LL, Wong CW, Chan KM, Li G, Waye MM, Zhang JF (2016). H19 activates Wnt signaling and promotes osteoblast differentiation by functioning as a competing endogenous RNA. Sci Rep.

[93] Wu N, Zhang H, Deng F, Li R, Zhang W, Chen X, Wen S, Wang N, Zhang J, Yin L, Liao Z, Zhang Z, Zhang Q (2014). Overexpression of Ad5 precursor terminal protein accelerates recombinant adenovirus packaging and amplification in HEK-293 packaging cells. Gene Ther.

[94] Chen X, Cui J, Yan Z, Zhang H, Chen X, Wang N, Shah P, Deng F, Zhao C, Geng N, Li M, Denduluri SK, Haydon RC (2015). Sustained high level transgene expression in mammalian cells mediated by the optimized piggyBac transposon system. Genes Dis.

[95] Deng Y, Wang Z, Zhang F, Qiao M, Yan Z, Wei Q, Wang J, Liu H, Fan J, Zou Y, Liao J, Hu X, Chen L (2016). A Blockade of IGF Signaling Sensitizes Human Ovarian Cancer Cells to the Anthelmintic Niclosamide-Induced Anti-Proliferative and Anticancer Activities. Cell Physiol Biochem.

[96] Liao Z, Nan G, Yan Z, Zeng L, Deng Y, Ye J, Zhang Z, Qiao M, Li R, Denduluri S, Wang J, Wei Q, Geng N (2015). The Anthelmintic Drug Niclosamide Inhibits the Proliferative Activity of Human Osteosarcoma Cells by Targeting Multiple Signal Pathways. Curr Cancer Drug Targets.

[97] He TC, Zhou S, da Costa LT, Yu J, Kinzler KW, Vogelstein B (1998). A simplified system for generating recombinant adenoviruses. Proc Natl Acad Sci U S A.

[98] Luo J, Deng ZL, Luo X, Tang N, Song WX, Chen J, Sharff KA, Luu HH, Haydon RC, Kinzler KW, Vogelstein B, He TC (2007). A protocol for rapid generation of recombinant adenoviruses using the AdEasy system. Nat Protoc.

[99] Li R, Yan Z, Ye J, Huang H, Wang Z, Wei Q, Wang J, Zhao L, Lu S, Wang X, Tang S, Fan J, Zhang F (2016). The Prodomain-Containing BMP9 Produced from a Stable Line Effectively Regulates the Differentiation of Mesenchymal Stem Cells. Int J Med Sci.

[100] Luo Q, Kang Q, Song WX, Luu HH, Luo X, An N, Luo J, Deng ZL, Jiang W, Yin H, Chen J, Sharff KA, Tang N (2007). Selection and validation of optimal siRNA target sites for RNAi-mediated gene silencing. Gene.

[101] Deng F, Chen X, Liao Z, Yan Z, Wang Z, Deng Y, Zhang Q, Zhang Z, Ye J, Qiao M, Li R, Denduluri S, Wang J (2014). A Simplified and Versatile System for the Simultaneous Expression of Multiple siRNAs in Mammalian Cells Using Gibson DNA Assembly. PLoS One.

[102] Zhao C, Wu N, Deng F, Zhang H, Wang N, Zhang W, Chen X, Wen S, Zhang J, Yin L, Liao Z, Zhang Z, Zhang Q (2014). Adenovirus-mediated gene transfer in mesenchymal stem cells can be significantly enhanced by the cationic polymer polybrene. PLoS One.

[103] Wen S, Zhang H, Li Y, Wang N, Zhang W, Yang K, Wu N, Chen X, Deng F, Liao Z, Zhang J, Zhang Q, Yan Z (2014). Characterization of constitutive promoters for piggyBac transposon-mediated stable transgene expression in mesenchymal stem cells (MSCs). PLoS One.

[104] Untergasser A, Cutcutache I, Koressaar T, Ye J, Faircloth BC, Remm M, Rozen SG (2012). Primer3—new capabilities and interfaces. Nucleic Acids Res.

[105] Lamplot JD, Liu B, Yin L, Zhang W, Wang Z, Luther G, Wagner E, Li R, Nan G, Shui W, Yan Z, Rames R, Deng F (2015). Reversibly Immortalized Mouse Articular Chondrocytes Acquire Long-Term Proliferative Capability while Retaining Chondrogenic Phenotype. Cell Transplant.

[106] Gao Y, Huang E, Zhang H, Wang J, Wu N, Chen X, Wang N, Wen S, Nan G, Deng F, Liao Z, Wu D, Zhang B (2013). Crosstalk between Wnt/beta-Catenin and Estrogen Receptor Signaling Synergistically Promotes Osteogenic Differentiation of Mesenchymal Progenitor Cells. PLoS One.

[107] Li Y, Wagner ER, Yan Z, Wang Z, Luther G, Jiang W, Ye J, Wei Q, Wang J, Zhao L, Lu S, Wang X, Mohammed MK (2015). The Calcium-Binding Protein S100A6 Accelerates Human Osteosarcoma Growth by Promoting Cell Proliferation and Inhibiting Osteogenic Differentiation. Cell Physiol Biochem.

[108] Wang J, Liao J, Zhang F, Song D, Lu M, Liu J, Wei Q, Tang S, Liu H, Fan J, Zou Y, Guo D, Huang J (2017). NEL-Like Molecule-1 (Nell1) Is Regulated by Bone Morphogenetic Protein 9 (BMP9) and Potentiates BMP9-Induced Osteogenic Differentiation at the Expense of Adipogenesis in Mesenchymal Stem Cells. Cell Physiol Biochem.

[109] Su Y, Wagner ER, Luo Q, Huang J, Chen L, He BC, Zuo GW, Shi Q, Zhang BQ, Zhu G, Bi Y, Luo J, Luo X (2011). Insulin-like growth factor binding protein 5 suppresses tumor growth and metastasis of human osteosarcoma. Oncogene.

[110] Ye J, Wang J, Zhu Y, Wei Q, Wang X, Yang J, Tang S, Liu H, Fan J, Zhang F, Farina EM, Mohammed MK, Zou Y (2016). A thermoresponsive polydiolcitrate-gelatin scaffold and delivery system mediates effective bone formation from BMP9-transduced mesenchymal stem cells. Biomed Mater.

[111] Wang J, Zhang H, Zhang W, Huang E, Wang N, Wu N, Wen S, Chen X, Liao Z, Deng F, Yin L, Zhang J, Zhang Q (2014). Bone Morphogenetic Protein-9 (BMP9) Effectively Induces Osteo/Odontoblastic Differentiation of the Reversibly Immortalized Stem Cells of Dental Apical Papilla. Stem Cells Dev.

[112] He BC, Chen L, Zuo GW, Zhang W, Bi Y, Huang J, Wang Y, Jiang W, Luo Q, Shi Q, Zhang BQ, Liu B, Lei X (2010). Synergistic antitumor effect of the activated PPARgamma and retinoid receptors on human osteosarcoma. Clin Cancer Res.

[113] Huang J, Bi Y, Zhu GH, He Y, Su Y, He BC, Wang Y, Kang Q, Chen L, Zuo GW, Luo Q, Shi Q, Zhang BQ (2009). Retinoic acid signalling induces the differentiation of mouse fetal liver-derived hepatic progenitor cells. Liver Int.

[114] Bi Y, He Y, Huang J, Su Y, Zhu GH, Wang Y, Qiao M, Zhang BQ, Zhang H, Wang Z, Liu W, Cui J, Kang Q (2014). Functional characteristics of reversibly immortalized hepatic progenitor cells derived from mouse embryonic liver. Cell Physiol Biochem.

