# Supplementary material for: lncRNA H19 mediates BMP9-induced osteogenic differentiation of mesenchymal stem cells (MSCs) through Notch signaling
Source: Oncotarget. 2017 Jun 27;8(32):53581–601. doi: 10.18632/oncotarget.18655 (PMC5581132; doi:10.18632/oncotarget.18655)
Supplement: Supplementary file 1 [file oncotarget-08-53581-s001.pdf]

lncRNA H19 mediates BMP9-induced osteogenic differentiation of mesenchymal stem cells (MSCs) through Notch signaling

SUPPLEMENTARY TABLE

Supplementary Table 1: List of the primers used for TqPCR

Gene/Transcript	qPCR Primer Sequence		Accession Number
	Forward	Reverse	
mouse Runx2	CCGGTCTCCTTCCAGGAT	GGGAACTGCTGTGGCTTC	NM_001146038.2
mouse Osx	GGGAGCAGAGTGCCAAGA	TACTCCTGGCGCATAGGG	NM_130458.3
mouse BSP	AGGGAACTGACCAGTGTTGG	ACTCAACGGTGCTGCTTTTT	NM_008318.3
mouse Ocn	CCAAGCAGGAGGGCAATA	TCGTCACAAGCAGGGTCA	NM_001305448.1
mouse Opn	CCTCCCGGTGAAAGTGAC	CTGTGGCGCAAGGAGATT	NM_001204201.1
mouse Gapdh	GCCTCGTCCCGTAGACAAAA	TTCCCATTCTCGGCCTTGAC	NM_008084
human NICD1	CCTGAGGGCTTCAAAGTGTC	CGGAACTTCTTGGTCTCCAG	NM_017617.4
mouse Notch1	CCCGCATTCCAACATCTC	GGTCCTGCATCCCACATC	NM_008714.3
mouse Notch2	AGCAGGAGGGGCAGGTAG	GGTTCGCTCAGCAGCATT	NM_010928.2
mouse Notch3	CTGGCTCCAGATGCCTGT	GGGGACAGCACCTCACAC	NM_008716.2
mouse Notch4	CCGTCCTGGTTTCACAGG	GACTTCCGTCAGGGCAGA	NM_010929.2
mouse Dll1	CCGGTTTGTGTGTGACGA	CCAGGGTCGCACATCTTC	NM_007865.3
mouse Dll3	GGGCTTCGATGTGAGGTG	GAAACCAGGTGGGCAATG	NM_007866.2
mouse Dll4	GGGCCTTCCTTCTGCATT	ACTCTTGGCGGGTTCACA	NM_019454.3
mouse Jag1	CCAACACGGTCCCCATTA	TTGGCAAAGCGGACTTTC	NM_013822.5
mouse Jag2	CACGCTGGCATGATCAAC	TGTTGCAGGTGGCACTGT	NM_010588.2
mmu-miR-106b	CCTGCTGGGACTAAAGTGCT	TACCCACAGTGCGGTAGC	MI0000407
mmu-miR-125a	CCCTTTAACCTGTGAGGACGT	GGCTCCCAAGAACCTCACC	MI0000151
mmu-miR-449b	AGACTCGGGTAGGCAGTGT	GTGGCAGGGTAGCTGTGG	MI0005547
mmu-miR-17	CAAAGTGCTTACAGTGCAGGT	GTGCCCTCACTGCAGTAGA	MI0000687
mmu-miR-449a	TGTGATGGCTTGGCAGTGT	TTAGCTGGTGCCGCTCAC	MI0001649
mmu-miR-34a	TGGCAGTGTCTTAGCTGGT	CAATGTGCAGCACTTCTAGGG	MI0000584
mmu-miR-107	TCAGCTTCTTTACAGTGTTGCC	AGCCCTGTACAATGCTGCT	MI0000684
mmu-miR-27b	AGGTGCAGAGCTTAGCTGA	GCCACTGTGAACAAAGCGG	MI0000142
mmu-miR-30a	GCCCCGGGCAAGAAGGCGAC	AGGCTGCAGGATGCTGCAGC	MI0000144
mmu-miR-351	TCCACTGCCTGGGTACATGG	AGACAAAGCAGCACGGAAGT	MI0000643
mmu-miR-128-1	CTGCACAGCACACAGGTTGG	GAGATTTATTTATGTGCTGA	MI0000155
mmu-miR-384	AAATGGAAACTGATTTGTTA	ATATCCATCAGGACATGTTA	MI0001146
mmu-miR-106a	GAGTATGCCTTGGCCATGTC	CCTTAACACAAGAGAATGTA	MI0000406
mmu-miR-34b	GACCCATACCTATTTGTGCT	CCTAGTAACTAGACTGTGCC	MI0000404
mmu-miR-30b	GGAAAAAGCAACATGCTAAG	TATGTTAACTGGTGGATACT	MI0000145
mmu-miR-103-1	TGTGCTGTTAATGAGTTCTT	AACTCGATTTATTTTGTCTC	MI0000587
